# Compassionate goals predict COVID-19 health behaviors during the SARS-CoV-2 pandemic

**DOI:** 10.1371/journal.pone.0255592

**Published:** 2021-08-06

**Authors:** Juan Ospina, Tao Jiang, Kennedy Hoying, Jennifer Crocker, Taylor Ballinger

**Affiliations:** Department of Psychology, The Ohio State University, Columbus, Ohio, United States of America; Middlesex University, UNITED KINGDOM

## Abstract

We predicted that people with compassionate goals to support others and not harm them practiced more COVID-19 health behaviors during the SARS-CoV-2 pandemic to protect both themselves and others from infection. Three studies (*N* = 1,143 American adults) supported these predictions and ruled out several alternative explanations. Compassionate goals unrelated to the health context predicted COVID-19 health behaviors better than the general motivation to be healthy (Studies 2 and 3). In contrast, general health motivation predicted general health behaviors better than did compassionate goals. Compassionate goals and political ideology each explained unique variance in COVID-19 health behaviors (Studies 1–3). Compassionate goals predict unique variance in COVID-19 health behaviors beyond empathic concern, communal orientation, and relational self-construal (Study 3), supporting the unique contribution of compassionate goals to understanding health behaviors. Our results suggest that ecosystem motivation is an important predictor of health behaviors, particularly in the context of a highly contagious disease.

## Introduction

SARS-CoV-2 is a highly contagious novel coronavirus that causes COVID-19, which can lead to serious illness and death. By June 15, 2021, more than 33 million people had been diagnosed and more than 600,000 people had died from COVID-19 in the United States [1]. To reduce the spread of SARS-CoV-2, public health officials recommended behaviors such as wearing a mask, washing hands, and social distancing [2]. Yet in the U.S., adherence to these guidelines has been inconsistent, and many people object to social distancing, mask wearing, and other health protective mandates [3, 4].

Why do some people follow health recommendations that could save lives, including their own, whereas others resist them? We propose that adherence to recommendations is explained, in part, by social motivations beyond the health context. Specifically, people with compassionate goals to be constructive and supportive and not harm others may practice COVID-19 health behaviors because they want to protect both themselves and others from infection.

### Theories of health behavior

A great deal of research and theory has examined predictors of health behaviors. Rational-choice based theories of health behavior, such as the health belief model, theory of reasoned action, and theory of planned behavior, explain health behaviors and behavioral intentions in terms of the value of health behaviors (benefits vs. costs for the individual) and expectancies regarding their likely success [5, 6 for reviews]. Social network theories also consider how other people influence health behaviors through social norms, culture, and social identities or structural and systemic constraints such as the availability and affordability of healthcare [7 for a review].

The SARS-CoV-2 pandemic has cast a spotlight on the importance of behaviors that can affect not only one’s own health, but also other people’s health. Existing theories of health behavior emphasize various influences on individuals’ decisions about behaviors that affect their own health. Models of contagious disease likewise emphasize behaviors that protect individuals from disease [8]. Prior to the SARS-CoV-2 pandemic, considerations regarding the effects of behaviors on *other people’s health* were largely absent from this work. These considerations may be particularly important in containing the spread of contagious diseases. Furthermore, most prior work focused on beliefs, motivations, and intentions regarding health behaviors specifically, while overlooking more general social motivations that may predict health behaviors.

Consistent with calls for social science research to guide responses to the SARS-CoV-2 pandemic [9], researchers have investigated personality characteristics and social motivations that predict recommended health behaviors during the pandemic [10]. For example, research has found that Australians higher in social focus values such as benevolence, conformity, and security are more willing to be vaccinated and report more social distancing [11]. The thought of infecting vulnerable people or large numbers of others can motivate social distancing behaviors [12]. Prosocial motivations and orientations predict likelihood of following health recommendations to reduce transmission of SARS-CoV-2 [13, 14]. In particular, people higher in empathic concern for others, and those who respond more emotionally to prosocial messages reported greater willingness to self-isolate and socially distance during the pandemic [15–17].

Thus, a growing body of research points to the importance of prosociality in predicting behaviors that prevent or reduce transmission of SARS-CoV-2. Existing theory and research typically conceptualizes prosociality as behaviors that benefit others but may incur costs to the self. In fact, most research and theory on prosocial orientations assumes that concern for one’s own well-being is self-centered or selfish, whereas concern for the well-being of others is selfless, or requires self-sacrifice [18]. In the context of highly contagious diseases, however, the well-being of oneself and the well-being of others are not mutually exclusive; prosocial motivation can integrate concern for one’s own and others’ well-being [19]. The present research uses egosystem-ecosystem theory as a framework to understand how social motivations to support others and not harm them, outside the health context, predict behaviors intended to slow or stop the transmission of the SARS-CoV-2 virus (i.e., COVID-19 health behaviors).

### Ecosystem motivation

According to egosystem-ecosystem theory, people have the capacity for two types of social motivation [20–22]. Drawing on biological ecosystems as a metaphor, people with ecosystem motivation view themselves as interconnected with all life and all people, including future and past generations and those in different physical locations [20]. They believe that the well-being of one person depends on the well-being of others and the interpersonal system as a whole. Ecosystem motivation relates to the caregiving or species-preservation system, and energizes behaviors that protect and support others’ well-being, but not at the expense of the self [20]. People with ecosystem motivation frame relationships in nonzero-sum ways, assuming that success and achievement are not zero-sum [20], and that problems can be resolved in ways that are good for themselves as well as others [23]. Accordingly, they have goals to promote the well-being of others in ways that are also good for themselves over time [20]. Crocker and Canevello [20] labeled these goals compassionate goals, which is something of a misnomer: compassionate goals are not the goal to have compassion for others’ suffering. Rather, people with compassionate goals want and try to be constructive and supportive and to not harm others, whether they are suffering or not. Compassionate goals predict prosocial intentions independent of emotional responses to others’ distress. Compassionate goals are the hallmark of ecosystem motivation [24].

Previous research supports these ideas [24 for a review]. For example, compassionate goals predict giving increased instrumental and emotional support to dyadic relationship partners over time. In turn, relationship partners notice this increased support and reciprocate, leading to virtuous cycles of support giving and receiving, as perceived by both partners [20, 25]. The finding that others’ notice and reciprocate the support given by people with compassionate goals validates the compassionate goals scale and demonstrates that the measure assesses more than socially desirable self-presentations. People with compassionate goals are particularly likely to give support that fits what others say they need and want [26].

Compassionate goals are theoretically and empirically distinct from several other measures of prosocial orientations, including empathic concern, communal orientation, and relational self-construals. The nonzero-sum thinking of people with compassionate goals (i.e., their tendency to view the well-being of others and themselves as interconnected) and their focus on intentions to behave in constructive and nonharmful ways represent unique aspects of ecosystem motivation compared to other measures of prosocial orientations. For example, empathic concern reflects an emotional response to others’ suffering that can motivate altruistic efforts to alleviate others’ distress [27]. Ecosystem motivation, in contrast, reflects a general intention to act in ways that are supportive, constructive and not harmful to others, whether they are in distress or not, and does not depend on emotional responses to others’ distress. Communal orientation is a norm that people in close relationships *should* respond to each other’s needs; communally oriented people are bothered when others do not respond to one’s own needs [28]. In contrast, compassionate goals reflect genuine concern with others’ well-being rather than a norm that people *should* respond to close others’ needs. Relational self-concepts refer to the tendency to include other people in mental representations of the self [29]. For people with relational self-concepts (also called relational-interdependent self-concepts), caring about close others is hard to distinguish from caring about the self. Accordingly, their prosocial behavior could be motivated by selfishness. In contrast, people with compassionate goals can see themselves as separate from others, yet understand how their well-being can be linked to that of others, including distant others and outgroups.

Consistent with these distinctions, research shows that compassionate goal items correlate moderately with other measures of prosocial orientations and consistently emerge as a separate factor in confirmatory analyses [26]. Furthermore, compassionate goals have unique explanatory power in predicting giving to others and the gratitude that recipients experience [26].

### Ecosystem motivation and health behaviors

We propose that compassionate goals predict health behaviors that prevent the spread of contagious diseases. Contagious diseases have an interpersonal dimension; they spread through social contact. Because people with compassionate goals want to promote the well-being of others as well as themselves, we hypothesize that compassionate goals may explain not only who adheres to recommendations to prevent the spread of contagious diseases, but also why they do so. Based on previous theory and research [22, 24], we propose that people higher in compassionate goals practice COVID-19 health behaviors more frequently. Furthermore, because they feel connected to others, we propose that they engage in COVID-19 health behaviors to protect their interpersonal ecosystem. Thus, they should report practicing COVID-19 health behaviors not only to protect themselves, but also close others and distant others, such as those in the broader community and around the world. Furthermore, because compassionate goals are theoretically and empirically distinct from several other types of prosocial orientations, we predict that they account for variability in COVID-19 health behaviors beyond what is accounted for by empathic concern, communal orientation, and relational self-concepts.

We predict that compassionate goals should associate more strongly with COVID-19 health behaviors than with general health behaviors. General health behaviors, such as eating a healthy diet and exercising regularly, can help protect individuals from many diseases such as heart disease, cancer, and diabetes [30]. These diseases are not contagious, so they may have fewer interpersonal consequences and therefore be less related to prosocial motivation than contagious diseases. We explored the association between ecosystem motivation and general health behaviors to test the idea that compassionate goals are particularly related to health behaviors that prevent the spread of contagious disease because these diseases have direct implications for both oneself and others.

### Egosystem motivation

When motivated by the egosystem, people view others as means or obstacles for satisfaction of their own needs and desires. They believe that people should take care of themselves, regardless of others’ needs and desires [20]. Accordingly, they strive to maintain, enhance, and protect desired images of themselves in their own and others’ eyes; they have self-image goals [20]. Egosystem motivation relates to the fight-or-flight or self-preservation system [31, 32] and energizes behaviors that benefit oneself without consideration for the well-being of others.

### Egosystem motivation and health behaviors

Our predictions concerning egosystem motivation and COVID-19 health behaviors were more tentative. People high in egoistic selfishness “are not concerned about the needs of either individuals or society but are single-mindedly centered on themselves” [33] and therefore have egosystem motivation [23]. Selfishness was related to stockpiling toilet paper in Serbia during the early stage of the pandemic [34]. Egoistically selfish people might be motivated to practice COVID-19 health behaviors in order to protect themselves, even as they are less concerned about infecting others. Alternatively, selfish people might prioritize activities with more immediate rewards than protecting health—their own or others’.

We also included a measure of self-image goals as an indicator of egosystem motivation [20]. Previous research found that impression management concerns are associated with unhealthy behaviors such as tanning and smoking [35 for a review]. Thus, self-image goals might predict fewer general health behaviors. We did not have hypotheses about their connection to COVID-19 health behaviors.

### Overview

Three studies tested our main hypotheses that people with compassionate goals report engaging in more COVID-19 health behaviors, and do so with the aim to protect themselves, close others, *and* distant others from infection. We also explored whether compassionate goals predict general health behaviors and whether egosystem motivation (self-image goals or egoistic selfishness) predicts COVID-19 or general health behaviors (Studies 1–3). Statistical mediation analysis using data from all three studies explored whether compassionate goals predicted more frequent COVID-19 health behaviors through the desire to protect oneself, close others, and distant others from infection.

Across the three studies we tested several potential alternative explanations that might account for associations between compassionate goals and COVID-19 health behaviors. Studies 1–3 controlled for gender, because women tend to be higher in compassionate goals [20] and also engage in more COVID-19 health behaviors [36]. Studies 1–3 also controlled for political ideology, which strongly predicts COVID-19 health behaviors in the U.S. [37], and could be correlated with compassionate goals. Studies 2 and 3 controlled for socially desirable response tendencies, which correlate with compassionate goals and might also predict self-reports of COVID-19 health behaviors. Studies 2–3 also examined general health motivation to test the possibility that people with compassionate goals practice COVID-19 health behaviors more because they have a stronger desire to be healthy. Finally, in Study 3, we tested whether compassionate goals predict unique variance in COVID-19 health behaviors and three reasons for those behaviors (protecting the self, close others, and distant others), when we controlled for other prosocial orientations including empathic concern, communal orientation, and relational self-construal.

## Study 1

### Methods

This research was reviewed and approved by the Behavioral and Social Sciences Institutional Review Board of the Ohio State University, study number 2019B0248, with waiver of written documentation of informed consent. All verbatim study materials, data files, cleaning and analysis scripts, power analyses, and supplementary materials for all studies are available via OSF: https://osf.io/uk35n/.

#### Participants and data quality

American adults (*N* = 402) recruited from Amazon.com’s MTurk via TurkPrime [38] received $1.50 for their participation ($7.85/hour calculated using the median time it took all participants to complete the study). Data collection occurred on June 9, 2020, when there were approximately 20,673 new daily-confirmed cases of the SARS-CoV-2 virus in the United States using the 7-day moving average [1]. All studies were approved by an Institutional Review Board.

Three participants were blocked from completing the study because they used a VPN/VPS, they responded from outside the United States, or both [39]. An additional 36 participants were excluded for failing at least one data quality check (see S1 Table). All analyses included 363 participants who reported their gender (51.0% male, 48.5% female, 0.5% non-binary), age (*M* = 36.91, *SD* = 11.46), race (75.8% non-Hispanic White, 9.1% Black or African-American, 6.1% East Asian, 3.6% Hispanic or Latino/a, 2.2% Multiracial, 1.4% South Asian, 0.8% Native American or First Nation, 0.3% Middle Eastern, and 0.8% another identity), and political ideology (52.1% liberal, 30.6% conservative, 17.4% independent or some other party). Using G*Power [40], a sensitivity power analysis revealed 80% power to detect effect sizes as small as *R*^*2*^ = .022 after controlling for gender, selfishness, and political ideology in a linear regression analysis.

#### Procedure

Participants learned that the study concerned their thoughts, feelings, and behaviors over the past month in reaction to the COVID-19 pandemic. After providing consent, participants completed the measures in the order described below. Additional measures collected for exploratory purposes are described in the S1 File. For each study, we conducted additional robustness checks after including these measures in our primary analyses. In general, our reported results remained largely unchanged after controlling for these additional measures (see S1 File). After completing the study, participants were debriefed and redirected to a separate survey to receive compensation.

#### Measures

Unless otherwise indicated, participants responded to each measure using a 5-point (1 = *Not at all*, 5 = *Extremely*) scale. Unstandardized means, standard deviations, reliabilities, and zero-order correlations are presented in Table 1.

**Table 1:** Descriptive statistics and zero-order correlations in Study 1.

Measure	1	2	3	4	5	6	7	8	9	10
(1) Compassionate goals	-									
(2) Self-image goals	.19***	-								
(3) Selfishness	-.44***	.08	-							
(4) General health behaviors	.24***	-.01	-.16**	-						
(5) COVID-19 health behaviors (CHB)	.46***	.02	-.32***	.22***	-					
(6) CHB—protect self	.22***	.07	-.08	-.01	.51***	-				
(7) CHB–protect close others	.30***	.05	-.23***	.06	.52***	.67***	-			
(8) CHB–protect distant others	.39***	.03	-.35***	.16**	.45***	.43***	.59***	-		
(9) Gender	.17**	.05	-.16**	-.04	.19***	.11*	.04	.10	-	
(10) Political ideology	-.08	.14**	.10	-.04	-.21***	-.11*	-.04	-.14**	-.03	-
Unstandardized mean	3.74	2.68	2.28	3.23	4.25	4.01	3.96	3.36	-	3.44
Standard deviation	0.81	0.88	0.84	0.81	0.66	1.17	1.06	1.20	-	1.90
Cronbach’s ⍺	.92	.89	.85	.81	.92	-	.81	.93	-	-

*Note*. *N* = 363. CHB = COVID-19 health behaviors; GHB = general health behaviors. All measures used a 5-point scale except gender (1 = *Male*, 2 = *Female* or *non-binary*) and political ideology (1 = *Strongly liberal*, 7 = *Strongly conservative*).

*p < .05

**p < .01

***p < .001.

*Interpersonal motivations*. Participants indicated their compassionate goals, self-image goals, and selfishness over the past month [20]. Considering the possibility that these motivations can change over time [25], and therefore may have changed due to the pandemic, we worded items so they referred to the past month to ensure that all participants were using the same time frame in their responses. The 8-item compassionate goals scale assessed ecosystem motivation (e.g., “I wanted or tried to be supportive of others” and “I wanted or tried to avoid doing anything that would be harmful to others”). The 9-item self-image goals scale assessed egosystem motivation (e.g., “I wanted or tried to avoid showing my weaknesses” and “I wanted or tried to get others to admire or respect me”). An eight-item measure of egocentric selfishness [33] was also included as an additional indicator of egosystem motivation (e.g., “when it came to helping myself or helping others, I tended to help myself” and “I have not been too concerned about what is best for society in general”).

*General and COVID-19 health behaviors*. To align with the motivation measures, measures of health behaviors also referred to the past month. Using a 5-point scale (1 = *Never True of Me*, 5 = *Always True of Me*), participants indicated how often they engaged in 24 health behaviors over the past month, including 16 COVID-19 health behaviors intended to slow or stop the transmission of the SARS-CoV-2 virus (e.g., “I wore a mask or face covering if I was around others in public” and “If I had to go out in public, I stayed at least 6 feet away from others”; [41]) and eight general health behaviors from the Wellness Behavior Inventory (e.g., “I ate healthy, well-balanced meals every day” and “I exercised for 20 continuous minutes or more, to the point of perspiration, at least 3 times per week”; [42]). A pilot study completed by 89 medical school students validated the distinction between COVID-19 health behaviors and general health behaviors; medical students rated the 16 COVID-19 health behaviors as more effective in preventing the spread of the virus relative to 8 general health behaviors (see S1 File for more detail).

*Reasons for engaging in COVID-19 health behaviors*. Participants rated how much they engaged in COVID-19 health behaviors to protect themselves (one item; “to protect yourself from being infected with COVID-19”), close others (three items; e.g., “to protect your family members you live with from being infected”), and distant others (three items, e.g., “to protect people in your community from being infected”) from infection.

*Political ideology*. We assessed political ideology because it predicts COVID-19 health behaviors [4]. Participants indicated their political ideology (liberal, conservative, or neither) and the strength of that identification (1 = *Slightly*, 2 = *Moderately*, 3 = *Strongly*). Responses were transformed into a 7-point scale (1 = *Strongly liberal*, 7 = *Strongly conservative*).

*Demographics*. Participants reported their gender identity, age, race (Black or African-American, East Asian, Latino/Latina, Middle Eastern, Native American or First Nation, Native Hawaiian or Pacific Islander, South Asian, White or European American, Multiracial, or Other) and ethnicity (Hispanic, Latino, or Latina: yes or no).

*Data quality checks*. Before providing consent, participants encountered a reCaptcha question [43]. Participants also answered one attention check item surreptitiously located within the questions that assessed loneliness, closeness to others, perceived stress, and life satisfaction: “I am reading this question and will select ‘Often’ as my answer.” At the conclusion of the study, participants were asked to identify a picture of an eggplant in order to identify non-American English speakers [39]. Participants were also asked to complete one Winograd schema [44]: “Mark yelled at Tim because he was angry. Who was angry?”.

### Results

As shown in Table 1, compassionate goals correlated positively with both COVID-19 health behaviors and general health behaviors and with all three reasons for engaging in COVID-19 health behaviors (protecting the self, close others, and distant others). Selfishness and political conservatism correlated negatively with COVID-19 health behaviors.

#### COVID-19 health behaviors

Multiple regression analyses examined whether compassionate goals predict unique variance in COVID-19 health behaviors. For each study, our reported results remained largely unchanged when using robust standard errors in our regression models (see S1 File).

All continuous predictor and outcome measures were standardized. For each study, the S1 File reports multicollinearity diagnostics for all regression models and additional robustness checks. These results did not reveal multicollinearity concerns in any study, VIFs < 2.64 (see S1 File).

In Model 1, gender and compassionate goals were entered as simultaneous predictors of COVID-19 health behaviors. Model 2 also added selfishness as a predictor and Model 3 also added political ideology. Self-image goals were not included in these models because they were not correlated with COVID-19 health behaviors; adding self-image goals in the model did not change the reported pattern of results (see S2 Table).

Compassionate goals predicted greater frequency of COVID-19 health behaviors in each model (see Table 2). Women reported more frequent COVID-19 health behaviors than men. Greater selfishness and more conservative ideology predicted less frequent COVID-19 health behaviors. Controlling for these variables minimally changed the association between compassionate goals and COVID-19 health behaviors, suggesting that they account for a different portion of the variance.

**Table 2:** Coefficients from multiple regression models predicting COVID-19 health behaviors in Study 1.

	Model 1			Model 2			Model 3		
Predictor	β	95% CI	*p*	β	95% CI	*p*	β	95% CI	*p*
Compassionate Goals	.44	[.35; .54]	< .001	.39	[.29; .49]	< .001	.38	[.28; .48]	< .001
Gender	.23	[.05; .42]	.015	.21	[.02; .39]	.027	.20	[.02; .39]	.027
Selfishness				-.13	[-.23; -.03]	.011	-.12	[-.22; -.02]	.020
Political Ideology							-.17	[-.26; -.08]	< .001
*R*^*2*^		.23			.24			.27	

*Notes*. *N* = 363 for all models. All regression coefficients are standardized. Gender was coded as 1 = *Male*, 2 = *Female* or *non-binary* and political ideology was coded as 1 = *Strongly liberal* and 7 = *Strongly conservative*.

#### Desire to protect oneself, close others, and distant others from COVID-19

Three multiple regression analyses examined whether people with more compassionate goals reported doing COVID-19 health behaviors because they wanted to protect themselves, close others, and distant others from COVID-19, after controlling for gender, selfishness, and political ideology. Compassionate goals predicted each of the three reasons in the hypothesized direction (see Table 3).

**Table 3:** Coefficients from multiple regression models predicting reasons for COVID-19 health behaviors in Study 1.

	Protect self			Protect close others			Protect distant others		
Predictor	β	95% CI	*p*	β	95% CI	*p*	β	95% CI	*p*
Compassionate Goals	.21	[.10; .32]	< .001	.24	[.13; .35]	< .001	.28	[.18; .39]	< .001
Gender	.16	[-.04; .37]	.112	-.03	[-.24; .17]	.740	.03	[-.16; .22]	.782
Selfishness	.04	[-.07; .15]	.505	-.13	[-.24; -.02]	.023	-.22	[-.32; -.11]	< .001
Political Ideology	-.09	[-.19; .01]	.075	-.004	[-.10; .10]	.936	-.10	[-.19; -.004]	.040
*R*^*2*^	.06			.10			.20			

*Notes*. *N* = 363 for all models. All regression coefficients are standardized. Gender was coded as 1 = *Male*, 2 = *Female* or *non-binary* and political ideology was coded as 1 = *Strongly liberal* and 7 = *Strongly conservative*.

#### General health behaviors

Using the same analysis strategy as for COVID-19 health behaviors, we also examined predictors of general health behaviors. In each model, compassionate goals predicted more general health behaviors (see Table 4), although the association appeared weaker compared to their association with COVID-19 health behaviors. Gender, selfishness, and political ideology did not significantly increase the variance explained in any of the models.

**Table 4:** Coefficients from multiple regression models predicting general health behaviors in Study 1.

	Model 1			Model 2			Model 3		
Predictor	β	95% CI	*p*	β	95% CI	*p*	β	95% CI	*p*
Compassionate Goals	.25	[.15; .35]	< .001	.22	[.11; .33]	< .001	.22	[.10; .33]	< .001
Gender	-.16	[-.36; .04]	.124	-.17	[-.38; .03]	.096	-.17	[-.38; .03]	.096
Selfishness				-.08	[-.19; .03]	.153	-.08	[-.19; .03]	.159
Political Ideology							-.01	[-.11; .09]	.823
*R*^*2*^		.06			.07			.07	

*Notes*. *N* = 363 for all models. All regression coefficients are standardized. Gender was coded as 1 = *Male*, 2 = *Female* or *non-binary* and political ideology was coded as 1 = *Strongly liberal* and 7 = *Strongly conservative*.

### Discussion

Study 1 supported our hypothesis that compassionate goals predict how frequently people practice COVID-19 health behaviors even after controlling for gender, egoistic selfishness, and political ideology (see S1 File and S3 Table for additional robustness checks). Also in line with our hypotheses, people with compassionate goals reported that they engaged in COVID-19 health behaviors because they want to protect themselves, close others, and distant others from infection.

Study 1 found that compassionate goals predicted general health behaviors, suggesting that people with compassionate goals might be more motivated to be healthy, which could account for their greater frequency of COVID-19 health behaviors. To address this possibility, Study 2 included a measure of general health motivation to test whether compassionate goals predict unique variance in COVID-19 health behaviors independent of the general motivation to be healthy. In addition, socially desirable response tendencies could inflate the association between compassionate goals and COVID-19 health behaviors. Although self-image goals to present oneself as having desirable qualities did not predict unique variance in COVID-19 health behaviors, Study 2 also included a measure of socially desirable responding.

## Study 2

### Methods

#### Participants and data quality

American adults (*N* = 408) recruited from TurkPrime received $1.50 for their participation ($8.11 /hour). Data collection occurred on September 2, 2020, when there were approximately 39,485 new daily-confirmed cases of COVID-19 in the United States using the 7-day moving average [1].

Seven participants were blocked from completing the study because they used a VPN/VPS, they responded from outside the United States, or both [39]. An additional 14 participants were excluded for failing at least one data quality check (see S4 Table). Thus, reported analyses are based on 387 participants who reported their gender (49.4% male, 50.6% female), age (*M* = 39.52, *SD* = 12.64), race (77.0% non-Hispanic White, 7.5% Black or African-American, 6.5% Hispanic or Latino/a, 4.7% East Asian, 3.1% Multiracial, 0.5% Native American or First Nation, 0.5% South Asian, and 0.3% Middle Eastern), and political ideology (53.2% liberal, 29.7% conservative, 17.1% independent or some other party). A sensitivity power analysis revealed 80% power to detect effect sizes as small as *R*^*2*^ = .02 after controlling for gender, political ideology, social desirability, general health motivation, and selfishness in a linear regression model.

#### Measures

All measures were identical to those in Study 1 unless otherwise noted. Unstandardized means, standard deviations, reliabilities, and correlations are presented in Table 5. As in Study 1, participants reported their compassionate and self-image goals, egocentric selfishness, COVID-19 health behaviors, and motivation to engage in these COVID-19 health behaviors to protect the self, close others, and distant others.

**Table 5:** Descriptive statistics and zero-order correlations in Study 2.

Measure	1	2	3	4	5	6	7	8	9	10	11	12	13	14	15
(1) Compassionate goals	-														
(2) Self-image goals	.21***	-													
(3) Selfishness	-.44***	.23***	-												
(4) COVID-19 health behaviors	.41***	.06	-.27***	-											
(5) CHB–protect self	.31***	.12*	-.17**	.61***	-										
(6) CHB–protect close others	.40***	.07	-.27***	.58***	.53***	-									
(7) CHB–protect distant others	.41***	.11*	-.27***	.53***	.45***	.62***	-								
(8) General health behaviors	.24***	-.10	-.20***	.21***	.18***	.15**	.16**	-							
(9) GHB–protect self	.39***	.13**	-.24***	.42***	.51***	.31***	.29***	.20***	-						
(10) GHB–protect close others	.46***	.14**	-.29***	.51***	.33***	.59***	.48***	.18***	.65***	-					
(11) GHB–protect distant others	.41***	.15**	-.26***	.48***	.29***	.46***	.70***	.18***	.55***	.80***	-				
(12) General health motivation	.29***	.05	-.23***	.25***	.21***	.17**	.16**	.58***	.34***	.33***	.29***	-			
(13) Social desirability	.31***	-.02	-.30***	.22***	.15**	.17**	.28***	.15**	.13*	.23***	.26***	.22***	-		
(14) Gender	.11*	-.03	-.10*	.17**	.10	.14**	.08	.02	.06	.08	.03	.04	.07	-	
(15) Political ideology	-.04	.04	-.02	-.24***	-.13**	-.18***	-.23***	-.06	-.14**	-.09	-.15**	.01	.13**	-.12*	-
Unstandardized mean	3.85	2.85	2.27	4.28	4.18	4.04	3.25	3.58	3.67	3.33	2.75	3.54	5.45	-	3.50
Standard deviation	0.70	0.86	0.88	0.66	1.03	1.05	1.24	0.75	1.13	1.30	1.29	1.04	3.31	-	1.93
Cronbach’s ⍺	.91	.89	.87	.92	-	.84	.94	.60	-	.91	.96	.96	.80	-	-

*Note*. *N* = 387. CHB = COVID-19 health behaviors; GHB = general health behaviors. All measures used a 5-point scale except social desirability (13-pt summed scale), gender (1 = *Male*, 2 = *Female* or *non-binary*), and political ideology (1 = *Strongly liberal*, 7 = *Strongly conservative*).

*p < .05

**p < .01

***p < .001

*General health motivation*. Because health motivation may have changed due to the pandemic, we worded items so they referred to the past month to ensure that all participants were using the same time frame in their responses. Using a 5-point scale (1 = *Not at all characteristic of me*, 5 = *Very characteristic of me*), participants answered 10 items assessing their general health motivation over the past month (e.g., “I was very motivated to be physically healthy” and “I tried to avoid engaging in behaviors that undermined my physical health” [45, 46]).

*General health behaviors*. General health behaviors were adapted from the Wellness Behavior Inventory [47]. Using a 5-point scale (1 = *Less than once a week or never*, 2 = *One day a week*, 3 = *2–3 days a week*, 4 = *4–5 days a week*, 5 = *Every day of the week*), participants responded to four items that assessed how often they engaged in different health behaviors over the past month (e.g., “I exercised for 20 continuous minutes or more” and “I ate fresh fruit and vegetables every day”).

*Reasons for engaging in general health behaviors*. Participants indicated how much they engaged in general health behaviors to protect themselves, close others, and distant others from getting sick from illness (see S1 File for results with these measures).

*Social desirability*. Participants responded to 13 true-false statements assessing their tendency to respond in socially desirable ways (e.g., “I’m always willing to admit when I make a mistake” and “I am always courteous, even to people who are disagreeable”; [48]). We calculated a composite score by taking the sum of desirable responses such that higher values indicated greater social desirability.

### Results

Compassionate goals correlated positively with COVID-19 health behaviors, general health behaviors, and all three reasons for engaging in COVID-19 health behaviors: to protect the self, close others, and distant others (see Table 5). General health motivation also correlated positively with COVID-19 health behaviors and general health behaviors.

#### COVID-19 health behaviors

As in Study 1, we conducted multiple regression analyses with COVID-19 health behaviors as our outcome. Model 1 included compassionate goals as a predictor and added gender and social desirability as covariates. Model 2 added general health motivation as a predictor. Model 3 added selfishness and Model 4 added political ideology.

As expected, compassionate goals predicted more frequent COVID-19 health behaviors in each model (see Table 6). General health motivation also predicted more frequent COVID-19 health behaviors, although the effect size was significantly weaker than the effect of compassionate goals in each model, 46.96 ≤ Wald χ^2^s(2) ≤ 65.51, *p*s < .001.

**Table 6:** Coefficients from multiple regression models predicting COVID-19 health behaviors in Study 2.

	Model 1			Model 2			Model 3			Model 4		
Predictor	β	95% CI	*p*	β	95% CI	*p*	β	95% CI	*p*	β	95% CI	*p*
Compassionate Goals	.36	[.27; .46]	< .001	.33	[.23; .43]	< .001	.31	[.20; .41]	< .001	.29	[.19; .39]	< .001
Gender	.25	[.06; .43]	.008	.24	[.06; .42]	.008	.24	[.06; .42]	.010	.18	[.004; .35]	.046
Social Desirability	.10	[.01; .20]	.039	.08	[-.01; .18]	.095	.07	[-.03; .17]	.154	.11	[.01; .20]	.024
General Health Motivation				.14	[.04; .23]	.005	.13	[.04; .23]	.007	.13	[.04; .22]	.006
Selfishness							-.07	[-.17; .04]	.209	-.07	[-.17; .03]	.163
Political Ideology										-.24	[-.33; -.15]	< .001
*R*^*2*^	.19			.21			.21			.27		

*Notes*. *N* = 387 for all models. All regression coefficients are standardized. Gender was coded as 1 = *Male*, 2 = *Female* or *non-binary* and political ideology was coded as 1 = *Strongly liberal* and 7 = *Strongly conservative*.

#### Desire to protect oneself, close others, and distant others from COVID-19

Three multiple regression analyses examined whether compassionate goals predict desire to protect the self, close others, and distant others from COVID-19, after controlling for gender, social desirability, general health motivation, selfishness, and political ideology. Compassionate goals predicted all three reasons in the expected direction (see Table 7).

**Table 7:** Coefficients from multiple regression models predicting reasons for COVID-19 health behaviors in Study 2.

	Protect self			Protect close others			Protect distant others		
Predictor	β	95% CI	*p*	β	95% CI	*p*	β	95% CI	*p*
Compassionate Goals	.23	[.12; .34]	< .001	.31	[.20; .41]	< .001	.30	[.20; .40]	< .001
Gender	.09	[-.10; .28]	.334	.13	[-.05; .32]	.152	-.02	[-.20; .16]	.832
Social Desirability	.06	[-.05; .16]	.267	.06	[-.04; .15]	.262	.19	[.10; .29]	< .001
General Health Motivation	.13	[.03; .23]	.011	.05	[-.05; .15]	.308	.01	[-.08; .10]	.818
Selfishness	-.02	[-.13; .09]	.738	-.10	[-.20; .01]	.063	-.09	[-.18; .02]	.094
Political Ideology	-.13	[-.22; -.03]	.009	-.18	[-.27; -.08]	< .001	-.25	[-.34; -.16]	< .001
*R*^*2*^	.13			.21			.26		

*Notes*. *N* = 387 for all models. All regression coefficients are standardized. Gender was coded as 1 = *Male*, 2 = *Female* or *non-binary* and political ideology was coded as 1 = *Strongly liberal* and 7 = *Strongly conservative*.

#### General health behaviors

Using the same regression approach as described for COVID-19 health behaviors, compassionate goals predicted more frequent general health behaviors (marginally significant in Model 2; see Table 8). Model 3 included self-image goals as a predictor because it correlated negatively with general health behaviors. General health motivation predicted general health behaviors more strongly than did compassionate goals in each model, 178.77 ≤ Wald χ^2^s(2) ≤ 180.34, *p*s < .001.

**Table 8:** Coefficients from multiple regression models predicting general health behaviors in Study 2.

	Model 1			Model 2			Model 3			Model 4		
Predictor	β	95% CI	*p*	β	95% CI	*p*	β	95% CI	*p*	β	95% CI	*p*
Compassionate Goals	.22	[.12; .32]	< .001	.08	[-.01; .17]	.071	.12	[.02; .22]	.016	.12	[.02; .22]	.024
Gender	-.03	[-.22; .17]	.772	-.03	[-.20; .14]	.722	-.04	[-.21; .12]	.598	-.06	[-22.; .11]	.482
Social Desirability	.09	[-.01; .19]	.092	.01	[-.08; .10]	.827	-.003	[-.09; .08]	.934	.01	[-.08; .10]	.878
General Health Motivation				.55	[.46; .64]	< .001	.55	[.46; .64]	< .001	.55	[.46; .64]	< .001
Self-image goals							-.15	[-.24; -.06]	.001	-.15	[-.24; -.06]	.001
Selfishness							.01	[-.09; .11]	.855	.01	[-.09; .11]	.902
Political Ideology										-.06	[-.14; .02]	.142
*R*^*2*^	.06				.34						.36						.36					

*Notes*. *N* = 387 for all models. All regression coefficients are standardized. Gender was coded as 1 = *Male*, 2 = *Female* or *non-binary* and political ideology was coded as 1 = *Strongly liberal* and 7 = *Strongly conservative*.

### Discussion

Study 2 replicated and extended the finding of Study 1 that compassionate goals predict how frequently people practice COVID-19 health behaviors and do so in order to protect themselves and others from the virus. As in Study 1, these associations were not explained by gender, egoistic selfishness, or political ideology (see S1 File and S6 Table for additional robustness checks including social norms and conspiracy beliefs). Study 2 also showed that these associations were not accounted for by social desirability or people’s general motivation to be healthy.

Of interest for the health motivation literature, the association between compassionate goals and COVID-19 health behaviors remained significant and largely unchanged when we controlled for the general motivation to be healthy, which also predicted COVID-19 health behaviors. Thus, compassionate goals and general health motivation each account for unique variance in COVID-19 health behaviors; the association between compassionate goals and COVID-19 health behaviors is not simply due to greater health motivation among people with more compassionate goals.

The results of Studies 1 and 2 demonstrate the importance of compassionate goals as a predictor of COVID-19 health behaviors. However, these studies do not address whether it does so beyond the effects of other prosocial orientations, such as empathic concern. We hypothesized that compassionate goals should be uniquely associated with COVID-19 health behaviors because, in contrast to other prosocial orientations, compassionate goals capture the general intention to support others and the self, regardless of whether others are in distress, without requiring an emotional response, and regardless of whether others are close or distant. To test this hypothesis, Study 3 included measures of empathic concern, communal orientation, and relational self-construal. Because the different forms of prosociality assessed by compassionate goals, empathic concern, communal orientation, and relational self-concept are correlated with each other [26], controlling for other measures of prosociality and additional covariates is a particularly stringent test of our hypotheses.

## Study 3

Study 3 tested four hypotheses preregistered at osf.io/gxvfe along with materials and analyses. Our hypotheses were 1) Compassionate goals predict greater frequency of COVID-19 health behaviors; 2) The relationship between compassionate goals and COVID-19 health behaviors remains statistically significant after controlling for gender, social desirability, general health motivation, selfishness, political ideology, communal orientation, empathic concern, and relational self-construal; 3) Compassionate goals predict greater desire to protect a) the self; b) close others; and c) distant others from infection with COVID-19; and 4) The associations predicted in hypothesis 3 remain statistically significant after controlling for gender, social desirability, general health motivation, selfishness, political ideology, communal orientation, empathic concern, and relational self-construal. All methods and analyses followed our preregistration plan unless noted as exploratory.

### Methods

#### Participants and data quality

American adults (*N* = 435) recruited from TurkPrime received $1.50 for their participation ($8.07/hour). Data collection occurred on December 14, 2020, when there were approximately 201,649 new daily-confirmed cases of COVID-19 in the United States using the 7-day moving average [1], and the U.S. had surpassed 300,000 COVID-19 deaths.

Two participants were blocked from completing the study because they used a VPN/VPS. An additional 40 participants were excluded for failing at least one data quality check (see S7 Table). All analyses included 393 participants who reported their gender (53.2% female, 46.1% male, 0.8% non-binary), age (*M* = 39.04, *SD* = 12.45), race (77.9% non-Hispanic White, 6.1% Black or African-American, 6.1% East Asian, 4.1% Hispanic or Latino/a, 3.8% Multiracial, 1.5% South Asian, and 0.5% Middle Eastern), and political ideology (52.2% liberal, 31.3% conservative, 16.5% independent or some other party). An a priori power analysis indicated that 395 participants would provide 80% power to detect an effect size between compassionate goals and COVID-19 health behaviors at least *R*^*2*^ = .022 after controlling for eight covariates in a regression model.

#### Measures

All measures were identical to Study 2 unless otherwise noted. Participants reported their compassionate and self-image goals over the past month, egocentric selfishness, general health motivation, social desirability, desire to practice general health behaviors to protect oneself, close others, and distant others, COVID-19 health behaviors, and desire to practice COVID-19 health behaviors to protect oneself, close others, and distant others. General health behaviors were assessed using the measure in Study 1 with slight modifications. We worded items for communal orientation, empathic concern, and relational self-concept so they referred to the past month to ensure that all participants were using the same time frame in their responses, to rule out the possibility that participants referred to different time frames when responding to measures, and thus increase our ability to compare their effects with those of compassionate goals.

*Communal orientation*. Using a 5-point scale (1 = *Not at all characteristic of me*, 5 = *Very characteristic of me*), participants completed 14 items to indicate their communal orientation over the past month (e.g., “I expected people I know to be responsive to my needs and feelings over the past month,” and “I believed people should go out of their way to be helpful”; [28]).

*Empathic concern*. Using a 5-point scale (1 = *Not at all characteristic of me*, 5 = *Very characteristic of me*), participants answered six items to report their empathic concern over the past month (e.g., “I often had tender, concerned feelings for people less fortunate than me” and “Other people’s misfortunes did not usually disturb me a great deal” reverse-scored; [49]).

*Relational self-construal*. Using a 5-point scale (1 = *Strongly disagree*, 5 = *Strongly agree*), participants responded to 11 items that assessed how much participants defined themselves in relation to close others over the past month (e.g., “My close relationships were a close reflection of who I am” and “If a person hurt someone close to me, I felt personally hurt as well”; [29]).

### Results

Unstandardized means, standard deviations, reliabilities, and correlations between measures are presented in Table 9.

**Table 9:** Descriptive statistics and zero-order correlations in Study 3.

Measure	1	2	3	4	5	6	7	8	9	10	11	12	13	14	15	16	17	18
1) Compassionate goals	-																	
2) Self-image goals	.18***	-																
3) Selfishness	-.39***	.25***	-															
4) Communal orientation	.56***	-.03	-.57***	-														
5) Empathic concern	.57***	-.09	-.62***	.72***	-													
6) Relational self-construal	.49***	.09	-.34***	.55***	.47***	-												
7) General health motivation	.26***	.14**	-.10	.15**	.13**	.16**	-											
8) Social desirability	.23***	-.04	-.30***	.16**	.23***	.02	.19***	-										
9) COVID-19 health behaviors	.41***	.02	-.34***	.35***	.44***	.30***	.19***	.12*	^-^									
10) CHB–protect self	.20***	.07	-.09	.16**	.21***	.13**	.14**	.03	.59***	-								
11) CHB–protect close others	.30***	.11*	-.23***	.29***	.30***	.28***	.20***	.08	.60***	.65***	-							
12) CHB–protect distant others	.33***	.16**	-.26***	.31***	.37***	.32***	.19***	.18***	.52***	.46***	.63***	-						
13) General health behaviors	.24***	.03	-.20***	.20***	.18**	.17**	.74***	.23***	.25***	.11*	.20***	.20***	-					
14) GHB–protect self	.14**	.14**	-.06	.07	.10*	.09	.35***	.08	.36***	.51***	.42***	.30***	.29***	-				
15) GHB–protect close others	.22***	.16**	-.18***	.16**	.17**	.21***	.26***	.14**	.39***	.35***	.63***	.43***	.23***	.60***	-			
16) GHB–protect distant others	.24***	.21***	-.17**	.19***	.24***	.29***	.18***	.19***	.36***	.25***	.42***	.70***	.17**	.43***	.71***	-		
17) Political ideology	.01	.02	.06	-.10	-.11*	.01	-.01	.15**	-.25***	-.24***	-.22***	-.25***	-.02	-.05	-.02	-.08	-	
18) Gender	.08	-.09	-.32***	.25***	.27***	.09	-.12*	.03	.15**	.09	.12*	.09	-.07	.02	.06	.03	-.01	-
Unstandardized mean	3.90	2.83	2.14	3.58	3.97	3.68	3.47	5.66	4.32	4.13	4.10	3.46	3.29	3.86	3.59	2.93	3.43	-
Standard deviation	0.68	0.86	0.86	0.58	0.91	0.75	1.14	3.20	0.65	1.15	1.09	1.23	0.86	1.16	1.27	1.35	1.99	-
Cronbach’s ⍺	.90	.89	.87	.79	.90	.92	.97	.79	.93	-	.89	.94	.83	-	.93	.96	-	

*Note*. *N* = 393. CHB = COVID-19 health behaviors; GHB = general health behaviors. All measures used a 5-point scale except social desirability (13-pt summed scale), gender (1 = *Male*, 2 = *Female* or *non-binary*), and political ideology (1 = *Strongly liberal*, 7 = *Strongly conservative*).

*p < .05

**p < .01

***p < .001.

#### Preregistered analyses

Consistent with our first hypothesis, compassionate goals predicted greater frequency of COVID-19 health behaviors, β = .41, *p* < .001, 95% CI [.32; .50]. Consistent with our second hypothesis, this association remained statistically significant in a regression analysis controlling for gender, social desirability, general health motivation, selfishness, and political ideology in Model 1, and adding communal orientation, empathic concern, and relational self-construal in Model 2 (see Table 10).

**Table 10:** Coefficients from multiple regression models predicting COVID-19 health behaviors in Study 3.

	Model 1			Model 2		
Predictor	β	95% CI	*p*	β	95% CI	*p*
Compassionate Goals	.31	[.21; .40]	< .001	.22	[.11; .34]	< .001
Gender	.16	[-.02; .34]	.087	.13	[-.05; .31]	.159
Social Desirability	.02	[-.08; .11]	.715	.02	[-.07; .11]	.657
General Health Motivation	.10	[.01; .19]	.026	.10	[.01; .19]	.028
Selfishness	-.16	[-.27; -.06]	.001	-.09	[-.20; .03]	.136
Political Ideology	-.25	[-.33; -.16]	< .001	-.24	[-.32; -.15]	< .001
Communal Orientation				-.09	[-.22; .05]	.209
Empathic Concern				.22	[.08; .36]	.002
Relational Self-Construal				.08	[-.02; .19]	.130
*R*^*2*^	.28			.30		

*Notes*. *N* = 393 for all models. All regression coefficients are standardized. Gender was coded as 1 = *Male*, 2 = *Female* or *non-binary* and political ideology was coded as 1 = *Strongly liberal* and 7 = *Strongly conservative*.

Consistent with our third hypothesis, compassionate goals predicted greater desire to protect the self (β = .20, *p* < .001, 95% CI [.10; .30]), close others (β = .30, *p* < .001, 95% CI [.20; .40]) and distant others (β = .34, *p* < .001, 95% CI [.24; .43]), from infection with COVID-19. Consistent with our fourth hypothesis, compassionate goals predicted greater desire to protect oneself, close others, and distant others after controlling for gender, social desirability, general health motivation, selfishness, political ideology in Model 1, and adding communal orientation, empathic concern, and relational self-construal in Model 2 (see Table 11).

**Table 11:** Coefficients from multiple regression models predicting reasons for COVID-19 health behaviors in Study 3.

	Protect self			Protect close others			Protect distant others		
Predictor	β	95% CI	*p*	β	95% CI	*p*	β	95% CI	*p*
Model 1									
Compassionate Goals	.18	[.08; .29]	.001	.22	[.12; .33]	< .001	.25	[.15; .35]	< .001
Gender	.20	[-.01; .40]	.056	.17	[-.02; .37]	.082	.09	[-.10; .28]	.359
Social Desirability	.02	[-.09; .12]	.746	.01	[-.09; .10]	.928	.12	[.03; .22]	.014
General Health Motivation	.11	[.01; .21]	.032	.15	[.05; .24]	.003	.10	[.01; .20]	.030
Selfishness	.04	[-.07; .15]	.465	-.09	[-.20; .02]	.091	-.09	[-.19; .02]	.106
Political Ideology	-.25	[-.35; -.16]	< .001	-.22	[-.31; -.13]	< .001	-.27	[-.36; -.18]	< .001
*R*^*2*^	.12			.18			.22		
Model 2									
Compassionate Goals	.13	[.01; .26]	.041	.13	[.01; .25]	.037	.12	[.004; .24]	.043
Gender	.18	[-.03; .38]	.089	.16	[-.04; .36]	.112	.06	[-.12; .25]	.504
Social Desirability	.02	[-.09; .12]	.747	.03	[-.07; .13]	.591	.15	[.05; .24]	.003
General Health Motivation	.11	[.01; .21]	.033	.14	[.04; .23]	.005	.09	[.001; .19]	.047
Selfishness	.09	[-.04; .22]	.171	-.03	[-.15; .10]	.662	.01	[-.11; .13]	.870
Political Ideology	-.24	[-.34; -.15]	< .001	-.22	[-.31; -.12]	< .001	-.26	[-.35; -.18]	< .001
Communal Orientation	-.05	[-.20; .10]	.526	.02	[-.13; .16]	.791	-.05	[-.18; .09]	.512
Empathic Concern	.14	[-.01; .29]	.071	.06	[-.09; .21]	.416	.17	[.03; .31]	.018
Relational Self-Construal	.04	[-.08; .16]	.545	.14	[.03; .26]	.014	.19	[.08; .30]	.001
*R*^*2*^	.13			.20			.26		

*Notes*. *N* = 392 for all models. All regression coefficients are standardized. Gender was coded as 1 = *Male*, 2 = *Female* or *non-binary* and political ideology was coded as 1 = *Strongly liberal* and 7 = *Strongly conservative*.

#### Exploratory analyses

*Additional predictors of COVID-19 health behaviors*. General health motivation and political ideology each explained unique variance in COVID-19 health behaviors beyond that explained by compassionate goals, consistent with previous results. In both models, compassionate goals predicted COVID-19 health behaviors better than general health motivation, 23.54 ≤ Wald χ^2^s(2) ≤ 54.19, *p*s < .001. Empathic concern, but not communal orientation or relational self-construal, also predicted unique variance in COVID-19 health behaviors.

*Predictors of general health behaviors*. Neither compassionate goals nor the other prosocial orientations predicted more frequent general health behaviors in regression analyses (see Table 12). General health motivation predicted more frequent and selfishness predicted less frequent general health behaviors.

**Table 12:** Coefficients from multiple regression models predicting general health behaviors in Study 3.

	Model 1			Model 2		
Predictor	β	95% CI	*p*	β	95% CI	*p*
Compassionate Goals	< |.01|	[-.08; .07]	.933	-.02	[-.11; .07]	.657
Gender	-.04	[-.18; .10]	.574	-.04	[-.19; .10]	.557
Social Desirability	.06	[-.01; .13]	.115	.06	[-.01; .14]	.084
General Health Motivation	.72	[.65; .79]	< .001	.71	[.64; 78]	< .001
Selfishness	-.12	[-.20; -.04]	.003	-.11	[-.20; -.02]	.022
Political Ideology	-.02	[-.09; .05]	.555	-.02	[-.09; .05]	.556
Communal Orientation				.05	[-.06; .15]	.381
Empathic Concern				-.03	[-.14; .08]	.593
Relational Self-Construal				.02	[-.06; .11]	.601
*R*^*2*^	.57			.57		

*Notes*. *N* = 393 for all models. All regression coefficients are standardized. Gender was coded as 1 = *Male*, 2 = *Female* or *non-binary* and political ideology was coded as 1 = *Strongly liberal* and 7 = *Strongly conservative*.

### Discussion

Results of Study 3 were consistent with our preregistered hypotheses. Despite much higher rates of COVID-19 in the US when Study 3 was conducted, the zero-order correlation between compassionate goals and COVID-19 health behaviors was identical to that found in Study 2, suggesting that people lower in compassionate goals were less likely to follow health recommendations even when the risk of contracting the disease was very high. Study 3 again found that compassionate goals predict COVID-19 health behaviors even controlling for several other known and potential predictors, including gender, socially desirable responding, selfishness, general health motivation, and political ideology, supporting the robustness of our findings (see S1 File and S9 Table for additional robustness checks).

In addition to testing whether our effects remained despite the increased prevalence of COVID-19, Study 3 also tested whether compassionate goals predict COVID-19 health behaviors even when we controlled for three other measures of prosocial orientations, with which it is strongly correlated. That is, we aimed to test whether compassionate goals predict unique variance in COVID-19 health behaviors beyond that explained by other measures of prosociality. Compassionate goals predicted unique variance in COVID-19 health behaviors and desire to protect the self, close others, and distant others, beyond that explained by our covariates and three other measures of prosociality, suggesting that compassionate goals capture a unique aspect of prosociality that may have important implications for understanding health motivation in the context of contagious disease.

### Combined analyses

Following the recommendations of Curren and Hussing [50], we combined our data from Studies 1–3 (*N* = 1,143) into a single dataset. Testing our hypotheses in this combined dataset maximizes statistical power and more accurately estimates effect sizes [51], although this method has been criticized by some [52]. Given the different rates of COVID-19 cases in the United States when the studies were conducted, it seemed possible that variance in our primary outcomes could be explained by the time of data collection. To test this possibility, we calculated the intraclass correlation coefficient (ICC), a ratio of between-group (in this case, between-study) variance relative to the sum of within- and between-group variance, for each of our primary outcomes. In other words, the ICC represents the proportion of outcome variance explained by between-study differences. The ICCs were less than .01 for COVID-19 health behaviors and each of the three reasons for engaging in COVID-19 health behaviors. Because this indicated minimal clustering by study, we did not account for a random effect of study in our combined analyses.

Regression analyses on the combined data included compassionate goals as a predictor and controlled for the covariates measured in all three studies: gender, selfishness, and political ideology. Compassionate goals predicted greater frequency of COVID-19 health behaviors. In addition, people with compassionate goals were more likely to say that they did those behaviors because they wanted to protect themselves, close others, and distant others from infection (see Tables 13 and 14). To test whether the associations of compassionate goals with the four outcomes were moderated by study, for each model, we included study as an additional predictor (two dummy coded variables) and the interactions between compassionate goals and the two dummy variables for study. None of the associations were moderated by study (*ps* > .10), indicating that the associations between compassionate goals and the outcomes were not different across the three studies.

**Table 13:** Coefficients from multiple regression models predicting COVID-19 health behaviors across three studies.

Predictor	β	95% CI	*p*
Compassionate Goals	.35	[.30; .41]	< .001
Gender	.18	[.08; .28]	.001
Selfishness	-.13	[-.19; -.08]	< .001
Political Ideology	-.21	[-.26; -.16]	< .001
*R*^*2*^		.26	

*Notes*. *N* = 1,143. All regression coefficients are standardized. Gender was coded as 1 = *Male*, 2 = *Female* or *non-binary* and political ideology was coded as 1 = *Strongly liberal* and 7 = *Strongly conservative*.

**Table 14:** Coefficients from multiple regression models predicting reasons for COVID-19 health behaviors across three studies.

	Protect self			Protect close others			Protect distant others		
Predictor	β	95% CI	*p*	β	95% CI	*p*	β	95% CI	*p*
Compassionate Goals	.23	[.17; .29]	< .001	.27	[.21; .33]	< .001	.30	[.24; .36]	< .001
Gender	.13	[.02; .25]	.024	.08	[-.03; .19]	.164	.02	[-.09; .12]	.741
Selfishness	.01	[-.06; .07]	.874	-.12	[-.18; -.06]	< .001	-.16	[-.21; -.10]	< .001
Political Ideology	-.15	[-.21; -.10]	< .001	-.13	[-.19; -.08]	< .001	-.19	[-.25; -.14]	< .001
*R*^*2*^	.09			.14			.20		

*Notes*. *N* = 1,142 for all models. All regression coefficients are standardized. Gender was coded as 1 = *Male*, 2 = *Female* or *non-binary* and political ideology was coded as 1 = *Strongly liberal* and 7 = *Strongly conservative*.

We conducted exploratory mediation analyses to test whether wanting to protect oneself, close others, and distant others simultaneously accounted for the association between compassionate goals and COVID-19 health behaviors. The present data are correlational and therefore cannot demonstrate a causal association among variables. However, our theoretical framework, supported by previous research, assumes that compassionate goals are causally prior to reasons for COVID-19 health behaviors (the mediators) and COVID-19 health behaviors (the outcome). Our theoretical framework supports the appropriateness of statistical mediation [53] although we acknowledge that other causal orderings of variables may also be consistent with the data.

**Fig 1:**
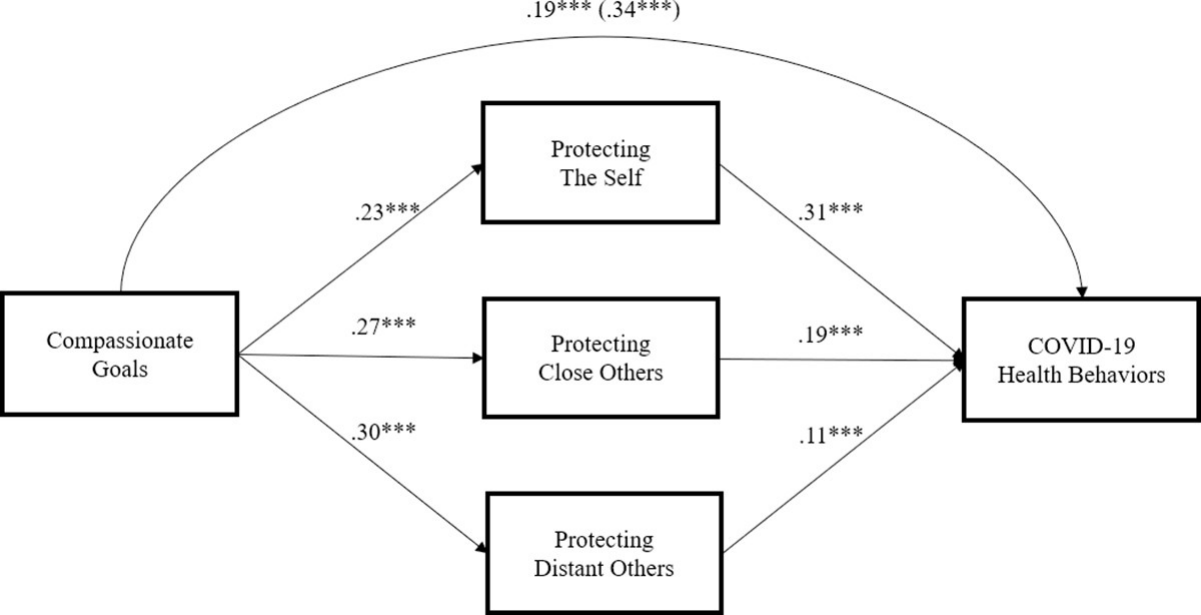
Mediation model controlling for gender, selfishness, and political ideology across three studies. All coefficients represent standardized effects. ***p < .001.

We used Model 4 in PROCESS [54] with 10,000 bootstrapped samples, with COVID-19 health behaviors as the outcome, compassionate goals as the predictor, the three reasons for engaging in COVID-19 health behaviors as parallel mediators, and gender, selfishness, and political ideology as covariates. Compassionate goals predicted COVID-19 health behaviors through each of the three reasons simultaneously (protecting the self: β = .07, 95% Boot CI [.05, .10]; protecting close others: β = .05, 95% Boot CI [.03, .08]; and protecting distant others: β = .03, 95% Boot CI [.01, .05]; see Fig 1). After accounting for these indirect effects, the direct effect remained statistically significant, β = .19, 95% CI = [.14, .23], *p* < .001. To test whether the indirect effects of compassionate goals on COVID-19 health behaviors through the three reasons depended on study, we used Model 59 in PROCESS with study as the moderator for all paths in the mediation model we tested. None of the three indirect effects was moderated by study (all 95% Boot CIs for indexes of moderated mediation contained 0), indicating that the indirect effects of compassionate goals on COVID-19 health behaviors through the three reasons were not different across the three studies.

### Discussion

Combining data sets is recommended because it affords more statistical power and more accurate estimates of effect sizes. After confirming that between-study differences on our primary outcome measures were minimal, we combined data into one data set. When we tested our main hypothesis that compassionate goals predict COVID-19 health behaviors in the data from all three studies combined, the predicted association was strong and highly significant. Furthermore, compassionate goals predicted people’s reports that they engaged in these behaviors because they wanted to protect themselves, close others, and distant others. These associations were highly significant despite controlling for other variables including gender, selfishness, and political orientation.

We also tested a mediation model in which compassionate goals predict COVID-19 health behaviors through the three reasons: to protect the self, close others, and distant others. Results indicated that the indirect effects of compassionate goals on COVID-19 health behaviors through each of the three reasons were statistically significant, consistent with the idea that people with compassionate goals engage in these behaviors because they want to protect themselves, close others, and distant others. Thus, each of the three reasons accounted for a portion of the association between compassionate goals and COVID-19 health behaviors. The direct effect was significant as well, suggesting that other mechanisms might also explain the association between compassionate goals and COVID-19 health behaviors. However, because the data were cross-sectional, we cannot rule out other causal associations among these variables.

## General discussion

Health behaviors such as social distancing and mask wearing have been among the most effective methods for preventing the spread of infection with SARS-CoV-2. Yet, even as the pandemic raged in the U.S., many people resisted behaviors that reduce the spread of infection. Prior to the SARS-CoV-2 pandemic, most theory and research on predictors of health behaviors focused on people’s motivations to protect their own health. The pandemic has focused attention on the interpersonal aspects of contagious diseases like COVID-19, and how social motivations may predict willingness to practice COVID-19 health behaviors. Specifically, a growing body of research has demonstrated that prosocial motivations and orientations predict people’s willingness to practice behaviors such as social distancing and wearing face coverings (e.g., 11–14, 16, 17].

Consistent with this prior research, the current studies point to the role of prosocial motivations as predictors of COVID-19 health behaviors. Three well-powered studies conducted at different phases of the pandemic in the US found that people with compassionate goals to be constructive and supportive of others in general report practicing COVID-19 health behaviors more frequently, and doing so because they want to protect not only themselves, but also close others such as friends and family, and distant others, such as people in their communities and in the world. The association between compassionate goals and COVID-19 health behaviors was strong and consistent at three very different phases of the SARS-CoV-2 pandemic, when the rate of infection in the US ranged from relatively low in June 2020, to when it was extremely high six months later. These results were not due to gender, socially desirable responding, or political ideology, which each also explained unique variance in COVID-19 health behaviors.

The present research builds on prior findings in several ways. First, they examine a type of prosocial motivation which, although studied extensively in other domains such as close relationships and psychological well-being [22, 24 for reviews], has not previously been examined in the context of health behaviors. Compassionate goals, the hallmark of ecosystem motivation, represent a distinct type of prosocial orientation in which the well-being of oneself and others are seen as interconnected and have nonzero-sum views of interpersonal relations. In the context of a highly contagious disease, we proposed that this form of prosociality might account for variation in practicing COVID-19 health behaviors.

Consistent with our theoretical perspective, people with compassionate goals reported that they practiced COVID-19 health behaviors more frequently because they wanted to protect themselves, close others, and distant others from infection with COVID-19. Exploratory analyses showed that these three reasons for engaging in COVID-19 health behaviors each simultaneously mediated the association between compassionate goals and COVID-19 health behaviors, consistent with the idea that people with compassionate goals view themselves as part of an interpersonal ecosystem in which the well-being of one depends on the well-being of others and the system as a whole. People with compassionate goals do not think that they must choose between protecting themselves or protecting others, and they want to protect distant others as much as they want to protect close others.

These associations remained significant in Study 3, when we controlled for empathic concern, communal orientation, and relational self-concept, providing further evidence that compassionate goals, although correlated with other measures, capture something unique about prosociality [26]. Indeed, compassionate goals was the only measure that significantly predicted unique variance in all three reasons for practicing COVID-19 health behaviors—protect the self, protect close others, and protect distant others. Two features of compassionate goals may explain their unique associations: people with compassionate goals want to promote the well-being of *both* themselves and others in a nonzero-sum way, and they want to do so even when others are not in distress, and are distant. These features may be particularly important in the SARS-CoV-2 pandemic, in which healthy-appearing people can be vulnerable to infection or even infectious themselves.

The present results also add to previous research by showing that compassionate goals predict COVID-19 health behaviors more strongly than they predict general health behaviors, such as eating a healthy diet and exercising. Furthermore, Studies 2 and 3 showed that the association between compassionate goals and COVID-19 health behaviors remained when we controlled for the general motivation to be healthy and avoid illness. These findings suggest that social motivations, and not just health motivations, may be important for understanding behavior related specifically to contagious diseases.

Also adding to existing research are findings on selfishness and health behaviors. When we combined data across three studies, egoistic selfishness predicted less frequent COVID-19 health behaviors separately from compassionate goals. However, this association may be due to less general health motivation among more selfish people; when general health motivation was controlled, selfishness no longer predicted COVID-19 health behaviors. Surprisingly, egoistic selfishness was unrelated to wanting to protect oneself from infection with SARS-CoV-2. As expected, egoistically selfish people were less motivated to protect close and especially distant others.

Studies on prosociality and COVID-19 health behaviors have been conducted in several countries. These behaviors have been highly politicized in the US, raising the possibility that political ideology could account for effects of compassionate goals. Consistent with prior research [37], political conservatism predicted less frequent COVID-19 health behaviors, independently of the effects of other predictors. More conservative people reported less desire to protect themselves, close others, and distant others from infection. Conservatives were not lower in general health behaviors or general health motivation, suggesting that their lower frequency of COVID-19 health behaviors could reflect skepticism about the seriousness of COVID-19. These results also add to existing findings by showing that political ideology was unrelated to compassionate goals; conservatives and liberals can and often do have compassionate goals toward other people in general.

### Implications for research on health motivation

These findings may have important implications for health behavior theories and research. Existing theories of health behavior focus on predicting behaviors that protect people’s own health, often in the context of noncontagious diseases such as diabetes, heart disease, or cancer. The extant research focuses on factors that affect health behavior intentions such as beliefs about the efficacy of health behaviors, expectancies about enacting them, availability of health resources, and so on [5].

The present findings suggest that the predictors of health behaviors may differ in the context of a pandemic, when one person’s behavior can also affect others’ health. Whereas compassionate goals predicted COVID-19 health behaviors better than did general health motivation, the reverse was true for behaviors that promote general health. These findings suggest that compassionate goals may be particularly important in the context of contagious diseases, when one’s behaviors have implications for both the self and other people.

### Practical implications

The SARS-CoV-2 pandemic has inspired research on what types of messages are most effective at increasing intentions to practice COVID-19 health behaviors [16, 36, 55, 56]. Research conducted prior to the pandemic demonstrated that other-focused messages are more effective than self-focused messages at promoting health behavior [57, 58]. In the context of the pandemic, the effectiveness of other-focused arguments for practicing COVID-19 health behaviors depends on characteristics of the message recipient [16, 56]. The present findings raise the possibility that messages that emphasize how people’s own behavior affects the health of others, and vice versa, may be particularly effective because they can activate the motivation to be healthy oneself and prosocial motivations in a nonzero-sum way. For example, as messaging encouraging vaccination becomes more targeted toward those who are hesitant, understanding and activating the compassionate goals of individual message recipients may be useful in persuasion.

### Limitations

The correlational methods used in these studies do not support causal inferences. For example, an alternative explanation of our data is that practicing more COVID-19 health behaviors increases people’s compassionate goals because people infer their goals from their behaviors. We attempted to reduce this possibility by asking questions about compassionate goals in general prior to asking about health behaviors, but we cannot rule it out entirely. Future research can address this limitation by using longitudinal or experimental methods to demonstrate the causal relationship between ecosystem motivation and health behaviors in the context of a contagious disease.

In addition, a methodological choice we made to assess motivational and behavioral constructs in terms of the past month might have affected the magnitude of the associations among these measures. We worded the items this way because prior research shows that social motivations can change over time [26]. Because the pandemic may have altered some people’s motivations, we wanted to ensure that all participants were thinking about the same time frame when responding to items. Putting all motivation and health behavior measures in the same temporal frame facilitates comparisons of their effects. However, using the same wording in these measures may have artifactually inflated correlations among them. This limitation should be addressed in future research.

Future research should also validate our findings with behavioral rather than self-report measures. In particular, because compassionate goals and COVID-19 health behaviors are both socially desirable, the tendency to respond in socially desirable ways could inflate their association. We addressed this possibility in Studies 2 and 3 and found that our effects remained when we controlled for socially desirable response tendencies. Although controlling for social desirability in this way does not entirely rule out this alternative explanation, we believe it is not a plausible account for our findings for several reasons. First, our measure of socially desirable responding is well-validated [34] and had good reliability. Second, we controlled for self-image goals to get others to see oneself as having desirable qualities in each of our studies. Third, several other measures that also correlated with socially desirable responding were also included in our analyses, so the remaining variance in COVID-19 health behaviors after controlling for those covariates is unlikely to be due to social desirability. Fourth, previous research has established that compassionate goals predict support and responsiveness as perceived by others [9, 12, 14], indicating that the compassionate goals scale is a valid measure of intentions to supportive and constructive.

Finally, although we found that compassionate goals account for unique variance in COVID-19 health behaviors, other measures of prosocial orientations might explain our findings. For example, readers might think that individualism-collectivism, which differs across cultures, might overlap with or account for the effects of compassionate goals. However, collectivism is a multifaceted distinction that cannot be reduced to compassionate goals [59]. Furthermore, measures of interdependence, which characterizes people in collectivist cultures, load on separate factors from items measuring compassionate goals [60]. Because of limits on participants’ time and attention, we could not include every plausible indicator of prosocial orientation in Study 3. Future research should continue to examine whether compassionate goals account for unique variance beyond other prosocial constructs.

## Conclusions

Nearly 600,000 people in the U.S. have died from COVID-19 and its complications. Although cases have waned in the US, they remain high in many parts of the world where people remain largely unvaccinated. Understanding what motivates adherence to COVID-19 health recommendations continues to be a matter of life and death. In the present studies, predictors of general health behaviors differed from predictors of COVID-19 health behaviors. Thus, these studies not only extend existing knowledge about who engage in health behaviors and why they engage in them, but also could help researchers, policy makers, and health practitioners to develop effective interventions to promote health behaviors for contagious diseases. Understanding the link between social motivations and health behaviors that prevent spread of contagious diseases may help save lives now and in the future.

## Supporting information

S1 Table Data quality checks in Study 1. (DOCX)

S2 Table Multiple regression models predicting COVID-19 health behaviors in Study 1. (DOCX)

S3 Table Multiple regression analyses predicting COVID-19 health behaviors and reasons for those behaviors in Study 1. (DOCX)

S4 Table Data quality checks in Study 2. (DOCX)

S5 Table Multiple regression models predicting reasons for general health behaviors in Study 2. (DOCX)

S6 Table Multiple regression analyses predicting COVID-19 health behaviors and reasons for those behaviors in Study 2. (DOCX)

S7 Table Data quality checks in Study 3. (DOCX)

S8 Table Multiple regression models predicting reasons for general health behaviors in Study 3. (DOCX)

S9 Table Additional robustness checks predicting COVID-19 health behaviors and reasons for those behaviors in Study 3. (DOCX)

S1 File (DOCX)
