# Supplementary material for: Compassionate goals predict COVID-19 health behaviors during the SARS-CoV-2 pandemic
Source: PLoS One. 2021 Aug 6;16(8):e0255592. doi: 10.1371/journal.pone.0255592 (PMC8345887; doi:10.1371/journal.pone.0255592)
Supplement: S1 Table — (DOCX) [file pone.0255592.s001.docx]

# Table S1. *Data quality checks in Study 1.*

| Quality Check | Pass | Fail | % Pass |
| --- | --- | --- | --- |
| VPN/VPS Use | 399 | 3 | 99.3% |
| ReCaptcha Test | 398 | 4 | 99.0% |
| Age Matches Birth Year | 379 | 23 | 94.3% |
| American English Speaker | 395 | 7 | 98.3% |
| Winograd Schema | 390 | 12 | 97.0% |
| **Passed All Quality Checks** | **363** | **39** | **90.3%** |

*Note.* Pass and fail columns indicate number of participants who passed and failed each quality check.
