# Supplementary material for: Compassionate goals predict COVID-19 health behaviors during the SARS-CoV-2 pandemic
Source: PLoS One. 2021 Aug 6;16(8):e0255592. doi: 10.1371/journal.pone.0255592 (PMC8345887; doi:10.1371/journal.pone.0255592)
Supplement: S2 Table — (DOCX) [file pone.0255592.s002.docx]

# Table S2. *Multiple regression models predicting COVID-19 health behaviors in Study 1*

|  | **Model 1** | | |  | **Model 2** | | |  | **Model 3** | | |
| --- | --- | --- | --- | --- | --- | --- | --- | --- | --- | --- | --- |
| **Predictor** | **β** | **95% CI** | ***p*** |  | **β** | **95% CI** | ***p*** |  | **β** | **95% CI** | ***p*** |
| Compassionate Goals | .44 | [.35, .54] | < .001 |  | .40 | [.30, .51] | < .001 |  | .39 | [.28, .49] | < .001 |
| Gender | .23 | [.05, .42] | .045 |  | .21 | [.03, .40] | .025 |  | .21 | [.02, .39] | .026 |
| Selfishness |  |  |  |  | -.12 | [-.22, -.02] | .022 |  | -.11 | [-.22, -.01] | .028 |
| Self-Image Goals |  |  |  |  | -.05 | [-.14, .05] | .308 |  | -.02 | [-.12, .07] | .632 |
| Political Ideology |  |  |  |  |  |  |  |  | -.16 | [-.25, -.07] | < .001 |
| *R^2^* |  | .23 |  |  |  | .24 |  |  |  | .27 |  |

*Notes*. All regression coefficients are standardized. Gender was coded as 1 = *Male*, 2 = *Female* or *non-binary* and political ideology was coded as 1 = *Strongly liberal* and 7 = *Strongly conservative.*
