# Supplementary material for: Compassionate goals predict COVID-19 health behaviors during the SARS-CoV-2 pandemic
Source: PLoS One. 2021 Aug 6;16(8):e0255592. doi: 10.1371/journal.pone.0255592 (PMC8345887; doi:10.1371/journal.pone.0255592)
Supplement: S3 Table — (DOCX) [file pone.0255592.s003.docx]

# Table S3. *Multiple regression analyses predicting COVID-19 health behaviors and reasons for those behaviors in Study 1*

|  | **COVID-19  health behaviors** | | |  | **Protect self** | | |  | **Protect close others** | | |  | **Protect distant others** | | |  |
| --- | --- | --- | --- | --- | --- | --- | --- | --- | --- | --- | --- | --- | --- | --- | --- | --- |
| **Predictor** | **β** | **95% CI** | ***p*** |  | **β** | **95% CI** | ***p*** |  | **β** | **95% CI** | ***p*** |  | **β** | **95% CI** | ***p*** | |
| Compassionate Goals | .38 | [.26, .49] | < .001 |  | .26 | [.13, .39] | < .001 |  | .24 | [.11, .37] | < .001 |  | .22 | [.10, .34] | < .001 | |
| Gender | .19 | [.01, .38] | .038 |  | .13 | [-.08, .33] | .214 |  | -.03 | [-.23, .17] | .753 |  | .04 | [-.15, .23] | .709 | |
| Selfishness | -.11 | [-.21, -.01] | .038 |  | .04 | [-.08, .15] | .516 |  | -.12 | [-.24, -.01] | .033 |  | -.19 | [-.30, -.08] | .001 | |
| Political Ideology | -.17 | [-.26, -.08] | < .001 |  | -.11 | [-.22, -.01] | .030 |  | < .01 | [-.10, .10] | .954 |  | -.08 | [-.17, .02] | .110 | |
| Age | .06 | [-.04, .15] | .232 |  | .12 | [.01, .22] | .029 |  | < .01 | [-.10, .11] | .941 |  | .02 | [-.08, .11] | .724 | |
| Nonzero-Sum Thinking | -.03 | [-.13, .07] | .550 |  | -.07 | [-.18, .04] | .220 |  | -.09 | [-.20, .02] | .104 |  | -.12 | [-.22, -.02] | .017 | |
| Mutual Satisfaction | .01 | [-.11, .14] | .817 |  | -.08 | [-.22, .06] | .257 |  | .05 | [-.09, .18] | .496 |  | .17 | [.04, .29] | .009 | |
| *R^2^* |  | .27 |  |  |  | .09 |  |  |  | .11 |  |  |  | .22 |  | |

*Notes*. All regression coefficients are standardized. Gender was coded as 1 = *Male*, 2 = *Female* or *non-binary* and political ideology was coded as 1 = *Strongly liberal* and 7 = *Strongly conservative.*
