# Supplementary material for: Compassionate goals predict COVID-19 health behaviors during the SARS-CoV-2 pandemic
Source: PLoS One. 2021 Aug 6;16(8):e0255592. doi: 10.1371/journal.pone.0255592 (PMC8345887; doi:10.1371/journal.pone.0255592)
Supplement: S5 Table — (DOCX) [file pone.0255592.s005.docx]

# Table S5. *Multiple regression models predicting reasons for general health behaviors in Study 2*

|  | **Protect self  from illness** | | | |  | **Protect close others  from illness** | | | | | | | | |  | **Protect distant others  from illness** | | | | | | | | |  |  |
| --- | --- | --- | --- | --- | --- | --- | --- | --- | --- | --- | --- | --- | --- | --- | --- | --- | --- | --- | --- | --- | --- | --- | --- | --- | --- | --- |
| **Predictor** | **β** | **95% CI** | ***p*** | | | |  | | **β** | | **95% CI** | | ***p*** | | | |  | | **β** | | **95% CI** | | ***p*** | | |  |
| Compassionate Goals | .29 | [.19, .39] | | < .001 | | | |  | | .35 | | [.25, .45] | | < .001 | | | |  | | .29 | | [.19, .39] | | < .001 | | |
| Gender | < .01 | [-.18, .18] | | .999 | | | |  | | .02 | | [-.16, .20] | | .827 | | | |  | | -.09 | | [-.27, .09] | | .342 | | |
| Social Desirability | -.01 | [-.11, .08] | | .765 | | | |  | | .07 | | [-.02, .17] | | .140 | | | |  | | .14 | | [.05, .24] | | .004 | | |
| General Health Motivation | .25 | [.15, .34] | | < .001 | | | |  | | .20 | | [.11, .29] | | < .001 | | | |  | | .17 | | [.08, .26] | | < .001 | | |
| Selfishness | -.06 | [-.16, .05] | | .271 | | | |  | | -.07 | | [-.16, .03] | | .192 | | | |  | | -.06 | | [-.16, .04] | | .248 | | |
| Political Ideology | -.13 | [-.22, -.04] | | .006 | | | |  | | -.09 | | [-.18, .002] | | .054 | | | |  | | -.16 | | [-.25, -.07] | | < .001 | | |

# *Notes*. All regression coefficients are standardized. Gender was coded as 1 (male) and 2 (female).
