# Supplementary material for: Compassionate goals predict COVID-19 health behaviors during the SARS-CoV-2 pandemic
Source: PLoS One. 2021 Aug 6;16(8):e0255592. doi: 10.1371/journal.pone.0255592 (PMC8345887; doi:10.1371/journal.pone.0255592)
Supplement: S6 Table — (DOCX) [file pone.0255592.s006.docx]

# Table S6. *Multiple regression analyses predicting COVID-19 health behaviors and reasons for those behaviors in Study 2*

|  | **COVID-19  health behaviors** | | |  | **Protect self** | | |  | **Protect close others** | | |  | **Protect distant others** | | |  |
| --- | --- | --- | --- | --- | --- | --- | --- | --- | --- | --- | --- | --- | --- | --- | --- | --- |
| **Predictor** | **β** | **95% CI** | ***p*** |  | **β** | **95% CI** | ***p*** |  | **β** | **95% CI** | ***p*** |  | **β** | **95% CI** | ***p*** | |
| Compassionate Goals | .26 | [.18, .34] | < .001 |  | .21 | [.12, .31] | < .001 |  | .29 | [.19, .38] | < .001 |  | .27 | [.18, .37] | < .001 | |
| Gender | .22 | [.08, .36] | .003 |  | .15 | [-.03, .32] | .095 |  | .15 | [-.02, .32] | .093 |  | -.04 | [-.21, .14] | .689 | |
| Social Desirability | .04 | [-.03, .12] | .284 |  | < .01 | [-.09, .10] | .932 |  | .01 | [-.08, .10] | .870 |  | .15 | [.06, .24] | .001 | |
| General Health Motivation | .13 | [.06, .20] | .001 |  | .12 | [.04, .21] | .006 |  | .05 | [-.04, .14] | .260 |  | .02 | [-.07, .11] | .657 | |
| Selfishness | -.02 | [-.10, .06] | .638 |  | .04 | [-.06, .14] | .416 |  | -.07 | [-.17, .03] | .146 |  | -.09 | [-.19, .01] | .077 | |
| Political Ideology | .01 | [-.07, .10] | .745 |  | .11 | [.01, .21] | .027 |  | -.01 | [-.10, .09] | .913 |  | -.16 | [-.26, -.06] | .002 | |
| Descriptive Norms | .02 | [-.05, .09] | .603 |  | .03 | [-.06, .11] | .539 |  | -.01 | [-.10, .07] | .766 |  | -.01 | [-.09, .08] | .880 | |
| Prescriptive Norms | .55 | [.45, .64] | < .001 |  | .43 | [.32, .54] | < .001 |  | .41 | [.30, .52] | < .001 |  | .35 | [.24, .46] | < .001 | |
| Conspiracy Beliefs | -.06 | [-.16, .03] | .202 |  | -.13 | [-.24, -.01] | .036 |  | -.01 | [-.12, .11] | .899 |  | .11 | [-.01, .23] | .070 | |
| *R^2^* |  | .54 |  |  |  | .34 |  |  |  | .35 |  |  |  | .34 |  | |

*Notes*. All regression coefficients are standardized. Gender was coded as 1 = *Male*, 2 = *Female* or *non-binary* and political ideology was coded as 1 = *Strongly liberal* and 7 = *Strongly conservative.*
