# Supplementary material for: Compassionate goals predict COVID-19 health behaviors during the SARS-CoV-2 pandemic
Source: PLoS One. 2021 Aug 6;16(8):e0255592. doi: 10.1371/journal.pone.0255592 (PMC8345887; doi:10.1371/journal.pone.0255592)
Supplement: S7 Table — (DOCX) [file pone.0255592.s007.docx]

# Table S7. *Data quality checks in Study 3.*

| Quality Check | Pass | Fail | % Pass |
| --- | --- | --- | --- |
| VPN/VPS Use | 433 | 2 | 99.5% |
| ReCaptcha Test | 434 | 1 | 99.8% |
| Age Matches Birth Year | 417 | 18 | 95.9% |
| American English Speaker | 431 | 4 | 99.1% |
| Attention Check | 419 | 16 | 96.3% |
| Winograd Schema | 425 | 10 | 97.7% |
| Passed All Quality Checks | 393 | 42 | 90.3% |

*Note.* Pass and fail columns indicate number of participants who passed and failed each quality check.
