# Supplementary material for: Compassionate goals predict COVID-19 health behaviors during the SARS-CoV-2 pandemic
Source: PLoS One. 2021 Aug 6;16(8):e0255592. doi: 10.1371/journal.pone.0255592 (PMC8345887; doi:10.1371/journal.pone.0255592)
Supplement: S8 Table — (DOCX) [file pone.0255592.s008.docx]

# Table S8. *Multiple regression models predicting reasons for general health behaviors in Study 3*

|  | **Protect self from illness** | | | |  | **Protect close others  from illness** | | | | | | | |  | **Protect distant others  from illness** | | | | | | | | |  |  |
| --- | --- | --- | --- | --- | --- | --- | --- | --- | --- | --- | --- | --- | --- | --- | --- | --- | --- | --- | --- | --- | --- | --- | --- | --- | --- |
| **Predictor** | **β** | **95% CI** | ***p*** | | | |  | | **β** | | **95% CI** | | ***p*** | | |  | | **β** | | | **95% CI** | ***p*** | | |  |
| Compassionate Goals | .04 | [-.09, .17] | | .525 | | | |  | | .09 | [-.04, .22] | .188 | | | | |  | | .05 | [-.08, .17] | | | .476 | | |
| Gender | .12 | [-.08, .33] | | .240 | | | |  | | .11 | [-.09, .32] | .283 | | | | |  | | .004 | [-.20, .21] | | | .972 | | |
| Social Desirability | .01 | [-.10, .11] | | .877 | | | |  | | .06 | [-.04, .17] | .230 | | | | |  | | .17 | [.06, .27] | | | .002 | | |
| General Health Motivation | .35 | [.25, .45] | | < .001 | | | |  | | .22 | [.11, .32] | < .001 | | | | |  | | .10 | [.00, .20] | | | .050 | | |
| Selfishness | .03 | [-.10, .16] | | .680 | | | |  | | -.08 | [-.21, .05] | .219 | | | | |  | | .01 | [-.12, .13] | | | .929 | | |
| Political Ideology | -.05 | [-.15, .04] | | .281 | | | |  | | -.03 | [-.13, .07] | .547 | | | | |  | | -.11 | [-.20, -.01] | | | .031 | | |
| Communal Orientation | -.07 | [-.22, .09] | | .393 | | | |  | | -.07 | [-.22, .08] | .352 | | | | |  | | -.10 | [-.25, .05] | | | .185 | | |
| Empathic Concern | .06 | [-.09, .21] | | .441 | | | |  | | -.003 | [-.16, .15] | .965 | | | | |  | | .11 | [-.05, .26] | | | .168 | | |
| Relational Self-Construal | .04 | [-.09, .16] | | .559 | | | |  | | .15 | [.03, .27] | .017 | | | | |  | | .26 | [.14, .38] | | | < .001 | | |

# *Notes*. All regression coefficients are standardized. Gender was coded as 1 (male) and 2 (female).
