# Supplementary material for: Compassionate goals predict COVID-19 health behaviors during the SARS-CoV-2 pandemic
Source: PLoS One. 2021 Aug 6;16(8):e0255592. doi: 10.1371/journal.pone.0255592 (PMC8345887; doi:10.1371/journal.pone.0255592)
Supplement: S9 Table — (DOCX) [file pone.0255592.s009.docx]

# Table S9. *Additional robustness checks predicting COVID-19 health behaviors and reasons for those behaviors in Study 3*

|  | **COVID-19  health behaviors** | | |  | **Protect self** | | |  | **Protect close others** | | |  | **Protect distant others** | | |  |
| --- | --- | --- | --- | --- | --- | --- | --- | --- | --- | --- | --- | --- | --- | --- | --- | --- |
| **Predictor** | **β** | **95% CI** | ***p*** |  | **β** | **95% CI** | ***p*** |  | **β** | **95% CI** | ***p*** |  | **β** | **95% CI** | ***p*** | |
| Compassionate Goals | .23 | [.12, .35] | < .001 |  | .14 | [.01, .27] | .032 |  | .12 | [-.01, .24] | .059 |  | .10 | [-.02, .22] | .090 | |
| Gender | .13 | [-.05, .32] | .154 |  | .18 | [-.03, .38] | .086 |  | .16 | [-.04, .35] | .117 |  | .06 | [-.13, .25] | .527 | |
| Social Desirability | .02 | [-.07, .11] | .653 |  | .02 | [-.09, .12] | .744 |  | .03 | [-.07, .13] | .594 |  | .15 | [.05, .24] | .003 | |
| General Health Motivation | .11 | [.02, .20] | .017 |  | .12 | [.02, .22] | .024 |  | .12 | [.03, .22] | .012 |  | .07 | [-.02, .16] | .125 | |
| Selfishness | -.09 | [-.21, .02] | .121 |  | .09 | [-.04, .22] | .187 |  | -.02 | [-.15, .10] | .718 |  | .02 | [-.10, .14] | .763 | |
| Political Ideology | -.24 | [-.32, -.15] | < .001 |  | -.25 | [-.34, -.15] | < .001 |  | -.22 | [-.31, -.12] | < .001 |  | -.26 | [-.35, -.17] | < .001 | |
| Communal Orientation | -.07 | [-.20, .07] | .348 |  | -.03 | [-.19, .12] | .687 |  | -.01 | [-.16, .14] | .921 |  | -.09 | [-.23, .05] | .201 | |
| Empathic Concern | .24 | [.10, .38] | .001 |  | .16 | [.002, .32] | .048 |  | .03 | [-.12, .18] | .680 |  | .12 | [-.02, .26] | .099 | |
| Relational Self-Construal | .09 | [-.02, .19] | .114 |  | .04 | [-.08, .16] | .511 |  | .14 | [.03, .25] | .017 |  | .18 | [.08, .29] | .001 | |
| Prosocial Intentions | -.08 | [-.19, .04] | .196 |  | -.06 | [-.19, .07] | .335 |  | .10 | [-.02, .23] | .110 |  | .17 | [.05, .29] | .005 | |
| *R^2^* |  | .30 |  |  |  | .13 |  |  |  | .20 |  |  |  | .28 |  | |

*Notes*. All regression coefficients are standardized. Gender was coded as 1 = *Male*, 2 = *Female* or *non-binary* and political ideology was coded as 1 = *Strongly liberal* and 7 = *Strongly conservative.*
