# Supplementary material for: Compassionate goals predict COVID-19 health behaviors during the SARS-CoV-2 pandemic
Source: PLoS One. 2021 Aug 6;16(8):e0255592. doi: 10.1371/journal.pone.0255592 (PMC8345887; doi:10.1371/journal.pone.0255592)
Supplement: S1 File — (DOCX) [file pone.0255592.s010.docx]

Compassionate Goals Predict COVID-19 Health Behaviors During the SARS-CoV-2

Pandemic

Supporting Information File

**Pilot Study**

**Overview**

In a pilot study, medical students were asked to evaluate to what extent various health behaviors would prevent the spread and transmission of the SARS-CoV-2 virus. These health behaviors varied in whether they were listed by the Centers for Disease Control and Prevention (CDC) as mitigation behaviors for preventing the spread of the SARS-CoV-2 virus (i.e., COVID-19 health behaviors) versus behaviors intended to promote a general healthy lifestyle (i.e., general health behaviors). We anticipated that medical students would rate the COVID-19 health behaviors as more effective in stopping the spread and transmission of the SARS-CoV-2 virus relative to the general health behaviors. We also expected that medical students would perceive the COVID-19 health behaviors as similarly effective in preventing the spread of the SARS-CoV-2 virus to both oneself as well as to other people.

**Methods**

Participants were 89 medical students at The Ohio State College of Medicine who voluntarily participated in the study. Among the 89 participants, 1.1% were in their first year of medical school, 30.3% were in their second year, 28.1% were in their third year, 32.6% were in their fourth year, and 7.9% were joint M.D./Ph.D. students who had completed two years of medical training and two years of research training.

Participants were contacted by email through a university listserv and were told that their responses would help identify “what behaviors are beneficial for preventing the infection and spread of COVID-19.” After providing informed consent, participants used a 5-point scale (1 = *Not at all*, 5 = *Extremely*) to rate 24 health behaviors on the extent to which they would “prevent someone from becoming infected [with the SARS-CoV-2 virus] themselves.” Of these 24 behaviors, 16 were intended to slow or stop the transmission of the SARS-CoV-2 virus (e.g., “practice social distancing by avoiding in-person social gatherings”) and eight were general health behaviors (e.g., “eat healthy, well-balanced meals everyday”).

Participants also rated these same 24 behaviors on the extent to which they would “help someone avoid infecting others [with the SARS-CoV-2 virus].” The order of these two measures was counterbalanced such that half of participants evaluated the effectiveness of each behavior for preventing the spread of the virus to the *self* before *others*, whereas the remaining participants evaluated the effectiveness of each behavior for preventing the spread of the virus to *others* before the *self*.

**Results**

The 16 COVID-19 health behaviors were internally reliable when considering the extent to which they prevented the self from becoming infected (⍺ = .81) and the extent to which they prevented others from becoming infected (⍺ = .82). Likewise, the eight general health behaviors were internally reliable when considering the extent to which they prevented the self from becoming infected (⍺ = .94) and the extent to which they prevented others from becoming infected (⍺ = .96).

We first examined whether medical students considered the COVID-19 health behaviors to be more effective protecting oneself from the SARS-CoV-2 virus relative to the general health behaviors. As expected, medical students perceived the COVID-19 health behaviors to be more effective in slowing the spread and transmission of the SARS-CoV-2 virus to the self (*M* = 4.34, *SD* = 0.44) relative to general health behaviors (*M* = 2.14, *SD* = 0.82), *t*(88) = 24.88, *p* < .001, *d*_z_ = 2.65. Likewise, medical students also perceived the COVID-19 health behaviors to be more effective in slowing the spread and transmission of the SARS-CoV-2 virus to others (*M* = 4.46, *SD* = 0.40) relative to general health behaviors (*M* = 1.72, *SD* = 0.84), *t*(88) = 28.11, *p* < .001, *d*_z_ = 2.97. This validated our primary outcome measure; the COVID-19 health behaviors were seen as more effective in preventing the spread of the virus, to both oneself and others, relative to general health behaviors.

We next examined whether medical students viewed the COVID-19 health behaviors to be more effective in slowing the spread and transmission of the SARS-CoV-2 virus to oneself versus others. Participants viewed the COVID-19 health behaviors to be more effective in slowing the spread of the SARS-CoV-2 virus to others (*M* = 4.46, *SD* = 0.40) versus to oneself (*M* = 4.34, *SD* = 0.44), *t*(88) = 4.18, *p* < .001, *d*_z_ = 0.43. However, these two measures were highly correlated, *r*(89) = .78, *p* < .001, indicating that participants viewed these behaviors to be similarly effective in both preventing the spread of the SARS-CoV-2 virus to oneself as well as others.

**Study 1**

**Supplemental Methods**

**Additional Measures**

Unless otherwise indicated, participants responded to each measure using a 5-point (1 = *Not at all*, 5 = *Extremely*) scale. The order of all measures was compassionate and self-image goals, egoistic selfishness, financial contingencies of self-worth, mutual satisfaction of needs, (non)zero-sum thinking, general and COVID-19 health behaviors, reasons for engaging in COVID-19 health behaviors, depression, anxiety, goal-related affect, loneliness, life satisfaction, closeness to others, perceived stress, emotional uncertainty, COVID-19 measures, and demographics. Below, we report all measures not described in the primary manuscript.

**Financial contingencies of self-worth.** Participants completed five items (⍺ = .82; e.g., “My self-esteem depends on having a lot of money”) assessing the extent to which their self-worth was rooted in financial success [1].

**Mutual satisfaction of needs.** Participants completed four items (⍺ = .65) that assessed how much they believed that their own needs could be met in collaboration with others during the past month (e.g., “In the past month, I thought both I and others could get what we needed”).

**(Non)Zero-sum thinking.** Participants responded to four items (⍺ = .84) that assessed how much they viewed social relations in nonzero-sum ways during the past month (e.g., “In the past month, I thought it was possible for everyone to get what they really needed”). Participants also answered four items (⍺ = .78) that assessed how much they viewed social relations in zero-sum terms during the past month (e.g., “In the past month, I focused on getting what I wanted even if it came at the expense of someone else”).

**Depression.** Using a 4-point scale (1 = *Rarely or none of the time, 2 = Some or little of the time*, 3 = *Moderately or much of the time,* 4 = *Most or almost all the time*), participants completed 20 items (⍺ = .95; e.g., “I felt depressed”) that assessed clinical symptoms of depression over the past month [3]. These items were summed to create a global composite of depressive symptoms.

**Anxiety.** Using a 5-point scale (1 = *Never*, 5 = *Always*), participants completed eight items (⍺ = .92; e.g., “I felt tense”) that assessed feelings of anxiety over the past month.

**Goal-related affect.** Using a 5-point scale (1 = *Never*, 5 = *Always*), participants completed 16 items measuring their goal-related affect over the past month [2]. Eight items (⍺ = .89) assessed feelings of emotional clarity (e.g., “I felt peaceful”) and eight items (⍺ = .85) assessed feelings of emotional confusion (e.g., “I felt conflicted”).

**Loneliness.** Using a 5-point scale (1 = *Never*, 5 = *Always*), participants answered three items (⍺ = .85) adapted from the Revised UCLA Loneliness Scale [4]: “I felt lonely”, “I was worried about whether others would accept me”, and “I was worried about being rejected or excluded by others.”

**Closeness to others.** Using a 5-point scale (1 = *Never*, 5 = *Always*), participants responded to four items (⍺ = .79) assessing how connected to others they felt over the past month: “I talked with someone about my emotions”, “I gave support to others”, “I received support from others”, and “I felt close to others.”

**Perceived stress.** Using a 5-point scale (1 = *Never*, 5 = *Always*), participants completed four items (⍺ = .81; e.g., “I felt I was unable to control the important things in my life) measuring their perceived stress over the past month [5].

**Life satisfaction.** Using a 5-point scale (1 = *Never*, 5 = *Always*), participants completed four items (⍺ = .93; e.g., “I felt life was very close to my ideal”) assessing how satisfied they felt with their life over the past month [6].

**Emotional uncertainty.** Participants completed six items (⍺ = .96) from the emotional uncertainty scale [7]. These items formed a composite such that higher values indicated a more maladaptive coping style in response to uncertainty (e.g., “I feel anxious when things are changing”).

**COVID-19 measures.** To assess perceived vulnerability to COVID-19, participants used a 5-point scale (1 = *Not at all*, 5 = *Extremely*) to respond to several statements: “My immediate family is vulnerable to contracting the COVID-19 virus due to location, occupation, etc.”, “My extended family is vulnerable to contracting the COVID-19 virus due to location, occupation, etc.”, and “My community is vulnerable to contracting the COVID-19 virus due to location, population density, etc.” To assess concerns about the COVID-19 virus and its impact on participants’ quality of life, participants used a 5-point scale (1 = *Not at all*, 5 = *Extremely*) to respond to several statements: “I am worried that if I get infected, I could transmit the coronavirus to someone that I care about”, “The COVID-19 pandemic has had a negative effect on my finances”, “The COVID-19 pandemic has had a negative effect on my emotional well-being”, and “The COVID-19 pandemic has had a negative effect on my social relationships.”

Participants also reported whether they were currently unemployed due to the COVID-19 pandemic, whether they have been working remotely during the COVID-19 pandemic, and whether someone in their immediate household has lost income due to the COVID-19 pandemic. To assess personal history with the COVID-19 virus, participants also indicated whether they have been previously infected with COVID-19, whether someone in their immediate household has been infected with COVID-19, and to what extent they viewed themselves and a member of their immediate household at risk of severe illness if they contracted the virus.

**Demographics.** Perceived socioeconomic status was measured using a 10-point ladder [8]. Participants also reported their annual gross household income, highest level of education, primary language, birth year, and ZIP code of primary residence.

**Supplemental Results**

**Data Quality**

Participants were excluded from all analyses if they failed one of several pre-defined data quality and attention check items. Table S1 describes the results of these data quality checks.

**Multicollinearity Diagnostics**

In Study 1, our analyses regressed each outcome on compassionate goals, participant gender, egoistic selfishness, and political ideology. We calculated the variance inflation factor (VIF) for each predictor when they were all entered simultaneously in the same model. The results showed that the VIF of compassionate goals was 1.25, gender was 1.03, egoistic

selfishness was 1.25, and political ideology was 1.01. These results revealed minimal multicollinearity concerns as all VIFs were below 10 [9].

**Do Compassionate Goals Predict More Frequent COVID-19 Health Behaviors?**

In addition to the reported results, we also conducted a series of multiple regression analyses while adding participants’ self-image goals as an additional covariate. Model 1 contained participant gender and compassionate goals as predictors of COVID-19 health behaviors. Model 2 added self-image goals and selfishness as predictors, and Model 3 added political ideology as a predictor.

The results are summarized in Table S2. In line with the reported results, compassionate goals predicted greater frequency of COVID-19 health behaviors in each model. Women reporting engaging in these behaviors more often than men. Greater selfishness and more conservative ideology also predicted less frequent COVID-19 health behaviors. Taken together, controlling for participants’ self-image goals did not substantively change the reported results.

**Additional Robustness Analyses**

**Additional covariates.** We conducted additional robustness checks to examine whether our reported results remained statistically significant after including additional covariate measures. We regressed participants’ COVID-19 health behaviors and the reasons for engaging in these behaviors on compassionate goals, gender, selfishness, political ideology, age, nonzero-sum thinking, and mutual satisfaction of needs. As shown in Table S3, compassionate goals predicted greater frequency of COVID-19 health behaviors and the reasons for engaging in those behaviors, even after controlling for these additional covariates. These findings suggest that our reported results could not be explained by nonzero-sum thinking, mutual satisfaction of needs, or participant age.

**Robust standard errors.** The White test [10] revealed heteroskedasticity in our reported regression models for COVID-19 health behaviors, $\chi$^2^(13, *N* = 363) = 36.85, *p* < .001, and engaging in COVID-19 health behaviors to protect the self, $\chi$^2^(13, *N* = 363) = 26.67, *p* = .014, and distant others, $\chi$^2^(13, *N* = 363) = 28.92, *p* = .007. Therefore, we also examined whether our reported results remained statistically significant after using robust standard errors in our regression analyses. Using the procedures outlined in the primary manuscript, we regressed each outcome on compassionate goals, participant gender, egocentric selfishness, and political ideology. After using robust standard errors, compassionate goals remained a significant predictor of COVID-19 health behaviors, β = .38, *SE* = .06, *p* < .001, 95% CI [.26, .50], and engaging in COVID-19 health behaviors to protect the self, β = .21, *SE* = .06, *p* < .001, 95% CI [.09, .32], and distant others from infection, β = .28, *SE* = .06, *p* < .001, 95% CI [.17, .40].

**Study 2**

**Supplemental Methods**

**Additional Measures**

Unless otherwise indicated, participants responded to each measure using a 5-point (1 = *Not at all*, 5 = *Extremely*) scale. The order of all measures was compassionate and self-image goals, selfishness, general health motivation, frequency of general health behaviors, reasons for

general health behaviors, frequency of COVID-19 health behaviors, reasons for COVID-19 health behaviors, social desirability, subjective health rating, pandemic fatigue, descriptive and prescriptive social norms, conspiracy beliefs, COVID-19 measures, and demographics. Below, we report all measures not described in the primary manuscript.

**Subjective health rating.** Using a 6-point scale (1 = *Very poor*, 6 = *Excellent*), participants responded to two items that assessed their physical health and emotional or mental health during the past month. Using a yes/no response option, participants also indicated whether their physical or emotional health limited their usual social activities with family or friends. If so, participants then used a 5-point scale (1 = *Not at all*, 5 = *Extremely*) to indicate to what extent.

**Pandemic fatigue.** Participants responded to six items (⍺ = .78) that assessed the extent to which they were tired of the consequences of the COVID-19 pandemic (e.g., “I am tired of following social distancing and stay-at-home guidelines”).

**Social norms.** Participants indicated their perceived social norms toward four different behaviors: “practicing social distancing”, “wearing a mask in public”, “avoiding non-essential travel”, and “staying at home as much as possible.” For each of these four behaviors, participants used a 100-point slider to indicate perceptions of descriptive norms (⍺ = .84; e.g., “what percent of Americans *have regularly* practiced social distancing”) and prescriptive norms (⍺ = .96; e.g., “what percent of Americans *should regularly* wear a mask in public”).

**Conspiracy beliefs.** Using a 5-point scale (1 = *Strongly disagree*, 5 = *Strongly agree*), participants responded to seven items (⍺ = .90) that assessed their endorsement of conspiracy beliefs about the COVID-19 virus (e.g., “The COVID-19 virus was created in a Chinese lab as a bioweapon”).

**COVID-19 measures.** To assess perceived vulnerability to the COVID-19 virus, participants used a 5-point scale (1 = *Not at all*, 5 = *Extremely*) to respond to the following statement: “My community is vulnerable to contracting the COVID-19 virus due to location, population density, etc.” To assess concerns about the COVID-19 virus and its impact on participants’ quality of life, participants used a 5-point scale (1 = *Not at all*, 5 = *Extremely*) to respond to several statements: “I am worried that if I get infected, I could transmit the coronavirus to someone that I care about”, “The COVID-19 pandemic has had a negative effect on my finances”, and “The COVID-19 pandemic has had a negative effect on my emotional well-being.”

Participants also reported whether they were currently unemployed due to the COVID-19 pandemic, whether they have been working remotely during the COVID-19 pandemic, and whether someone in their immediate household had lost income due to the effects of the COVID-19 pandemic. To assess personal history with the COVID-19 virus, participants also indicated whether they have been previously infected with the COVID-19 virus, whether someone in their immediate household has been infected with the COVID-19 virus, and to what extent they viewed themselves and a member of their immediate household at risk of severe illness from the COVID-19 virus.

**Data quality.** Participants completed the same data quality checks and measures described in Study 1. In Study 2, the attention check item read “If you are reading this statement, please select ‘A little’ as your answer’”. This attention check item was surreptitiously embedded within the pandemic fatigue measure.

**Supplemental Results**

**Data Quality**

Participants were excluded from all analyses if they failed one of several pre-defined data quality and attention check items. Table S4 describes the results of each data quality check in Study 2.

**Multicollinearity Diagnostics**

In Study 2, our reported analyses regressed each outcome on compassionate goals, participant gender, social desirability, general health motivation, egoistic selfishness, and political ideology. We calculated the VIF for each predictor when they were all entered simultaneously in the same model. The results showed that the VIF of compassionate goals was 1.36, participant gender was 1.03, social desirability was 1.19, general health motivation was 1.12, selfishness was 1.31, and political ideology was 1.04. These results again revealed minimal multicollinearity concerns as all VIFs were below 10.

**Reasons for Engaging in General Health Behaviors**

We conducted multiple regression analyses to assess the extent to which our predictors were related to participants’ desire to protect themselves, close others, and distant others from general illness. In three separate regression analyses, we regressed each reason simultaneously on participants’ compassionate goals, gender, social desirability, general health motivation, selfishness, and political ideology. The results are displayed in Table S5. In general, both compassionate goals and the general motivation to be healthy predicted greater desire to practice healthy behaviors in order to protect oneself, close others, and distant others from illness.

We also conducted a multiple regression analysis to assess whether participants’ desire to protect themselves, close others, and distant others from illness predicted general health behaviors. We regressed general health behaviors on these three reasons simultaneously. Only desire to protect oneself from illness predicted more frequent general health behaviors and

protecting the self: β = .13, *p* = .042, 95% CI [.01, .26]; protecting close others: β = .03, *p* = .785, 95% CI [-.15, .20]; and protecting distant others from general illness: β = .09, *p* = .280, 95% CI [-.07, .25]).

**Additional Robustness Analyses**

**Additional covariates.** We conducted additional robustness analyses to examine whether our reported results remained statistically significant after including additional covariates. In multiple regression analyses, we regressed participants’ COVID-19 health behaviors and the reasons for engaging in these behaviors on compassionate goals, gender, social desirability, general health motivation, selfishness, political ideology, descriptive norms about COVID-19 health behaviors, prescriptive norms about COVID-19 health behaviors, and endorsement of conspiracy beliefs. As shown in Table S6, compassionate goals remained a significant predictor of COVID-19 health behaviors and each of the three reasons for engaging in these behaviors, even after adding these additional control variables. These findings suggest that the reported relationship between compassionate goals and COVID-19 health behaviors could not be explained by descriptive norms, prescriptive norms, or conspiracy beliefs about the virus.

**Robust standard errors.** The White test [10] revealed heteroskedasticity in our reported regression models for engaging in COVID-19 health behaviors to protect the self, $\chi$^2^(26, *N* = 387) = 67.41, *p* < .001, close others,$\chi$^2^(26, *N* = 387) = 46.12, *p* = .009, and distant others from infection, $\chi$^2^(26, *N* = 387) = 44.13, *p* = .015. Therefore, we also examined whether our reported results remained statistically significant after using robust standard errors in our regression analyses. Using the procedures outlined in the primary manuscript, we regressed each outcome on compassionate goals, participant gender, social desirability, general health motivation, egocentric selfishness, and political ideology. After using robust standard errors, compassionate

Goals remained a significant predictor of COVID-19 health behaviors, β = .29, *SE* = .06, *p* < .001, 95% CI [.18, .40], and engaging in COVID-19 health behaviors to protect the self, β = .23, *SE* = .07, *p =* .001, 95% CI [.10, .36], close others, β = .31, *SE* = .06, *p* < .001, 95% CI [.19, .43], and distant others from infection, β = .30, *SE* = .06, *p* < .001, 95% CI [.19, .41].

**Study 3**

**Supplemental Methods**

**Additional Measures**

Unless otherwise indicated, participants responded to each measure using a 5-point (1 = *Not at all*, 5 = *Extremely*) scale. Participants first completed measures of self-image and compassionate goals. Next, participants completed the following blocks of measures in a randomized order: communal orientation, empathic concern, relational self-construal, prosocial behavioral intentions, selfishness, general health motivation, and social desirability. Afterwards, participants reported their general health behaviors and the reasons for those behaviors (to

protect oneself, close others, and distant others from illness) and their COVID-19 health

behaviors and the reasons for those behaviors (to protect oneself, close others, and distant others from infection with the SARS-CoV-2 virus). The order of these two measures was counterbalanced such that half of participants completed the general health behaviors and reasons measures before the COVID-19 health behaviors and reasons measures, and vice-versa. Participants then answered several questions about COVID-19, provided their demographics and answered data quality questions, and were debriefed and dismissed.

**Prosocial behavioral intentions scale**. Using Baumsteiger & Siegel’s [11] measure, participants used a 5-point scale (1 = *Definitely would NOT do this,* 5 = *Definitely would do this*) to complete four items (⍺ = .86) that assessed their likelihood of engaging in several prosocial behaviors (e.g., “Help a stranger find something they lost, like their key or a pet”).

**Subjective health rating.** Using a 6-point scale (1 = *Very poor*, 6 = *Excellent*), participants responded to two items that assessed their physical health and emotional or mental health during the past month. Using a yes/no response option, participants also indicated whether their physical or emotional health limited their usual social activities with family or friends. If so, participants then used a 5-point scale (1 = *Not at all*, 5 = *Extremely*) to indicate to what extent.

**COVID-19 measures.** Participants used a 5-point scale (1 = *Not at all*, 5 = *Extremely*) to indicate their willingness to get the COVID-19 vaccine and their eagerness to receive the vaccine. Participants also completed the same COVID-19 measures described in Studies 1 and 2.

**Supplemental Results**

**Data Quality**

Participants were excluded from all analyses if they failed one of several pre-defined data quality and attention check items. Table S7 describes the results of each data quality check in Study 3.

**Multicollinearity Diagnostics**

In Study 3, our primary analyses regressed each outcome on compassionate goals, participant gender, social desirability, general health motivation, egoistic selfishness, political ideology, communal orientation, empathic concern, and relational self-construal. We calculated the variance inflation factor (VIF) for each predictor when they were all entered simultaneously in the same model. The results showed that the VIF of compassionate goals was 1.79, gender was 1.18, social desirability was 1.23, general health motivation was 1.13, selfishness was 1.89, communal orientation was 2.56, empathic concern was 2.64, and relational self-construal was

1.60. These results demonstrated minimal concerns for multicollinearity between predictors in Study 3, as all VIFs were below 10.

**Reasons for Engaging in General Health Behaviors**

In Study 3, we conducted multiple regression analyses to assess the extent to which our predictors were related to participants’ desire to protect themselves, close others, and distant others from illness. In three separate regression analyses, we regressed each reason simultaneously on participants’ compassionate goals, gender, social desirability, general health motivation, selfishness, political ideology, communal orientation, empathic concern, and relational self-construal. The results are displayed in Table S8. Compassionate goals did not predict any of the three reasons, whereas general health motivation predicted greater desire to protect oneself and close others from illness, and relational self-construal predicted greater desire to protect close and distant others from illness.

We also conducted a multiple regression analysis to assess whether participants’ desire to protect themselves, close others, and distant others from illness predicted general health behaviors. We regressed general health behaviors on the three reasons simultaneously. As in Study 2, only desire to protect oneself from illness predicted more frequent general health behaviors (protecting the self: β = .22, *p* < .001, 95% CI [.11, .34]; protecting close others: β = .08, *p* = .316, 95% CI [-.07, .22]; and protecting distant others: β = .02, *p* = .822, 95% CI [-.12, .15]).

**Additional Robustness Analyses**

**Additional covariates.** We conducted additional robustness analyses to examine whether our reported results remained statistically significant after including prosocial behavioral intentions in our analyses. In multiple regression analyses, we regressed participants’ COVID-19 health behaviors and the reasons for engaging in these behaviors on compassionate goals, participant gender, social desirability, general health motivation, egoistic selfishness, political ideology, communal orientation, empathic concern, relational self-construal, and prosocial behavioral intentions.

As shown in Table S9, compassionate goals remained a significant predictor of COVID-19 health behaviors after adding prosocial behavioral intentions in the model. Compassionate goals also remained a significant predictor of practicing COVID-19 health behaviors in order to protect the self from the virus, and a marginal predictor of practicing COVID-19 health behaviors in order to protect close and distant others from infection. These findings suggested that prosocial behavioral intentions could not account for the relationship between compassionate goals and frequency of COVID-19 health behaviors. However, prosocial behavioral intentions did account for participants’ reasons for practicing those behaviors, mainly to prevent both close and distant others from becoming infected.

**Robust standard errors.** The White test [10] revealed heteroskedasticity in our reported regression models for frequency of COVID-19 health behaviors, $\chi$^2^(52, *N* = 393) = 123.39, *p* < .001, and engaging in COVID-19 health behaviors to protect close others from infection, $\chi$^2^(52, *N* = 393) = 87.37, *p* = .002. Therefore, we also examined whether our reported results remained statistically significant after using robust standard errors in our regression analyses. Using the procedures outlined in the primary manuscript, we each outcome on compassionate goals, participant gender, social desirability, general health motivation, egocentric selfishness, political ideology, communal orientation, empathic concern, and relational self-construal.

After using robust standard errors, compassionate goals remained a significant predictor of COVID-19 health behaviors, β = .22, *SE* = .07, *p* = .003, 95% CI [.08, .37]. However, when

using robust standard errors, compassionate goals became a marginally-significant predictor of engaging in COVID-19 health behaviors to protect close others, β = .12, *SE* = .07, *p =* .087, 95% CI .-.02, .26].

**Supplemental References**

1. Park LE, Ward DE, Naragon-Gainey K. It’s all about the money (for some): Consequences of Financially Contingent Self-Worth. Personality and Social Psychology Bulletin. 2017;43(5):601–22.
2. Niiya Y, Crocker J, Mischkowski D. Compassionate and Self-Image Goals in the United States and Japan. Journal of Cross-Cultural Psychology. 2013 Apr 1;44(3):389–405
3. Radloff LS. The CES-D Scale: A self-report depression scale for research in the general population. Applied Psychological Measurement. 1977;1(3):385–401.
4. Russell D, Peplau LA, Cutrona CE. The revised UCLA Loneliness Scale: Concurrent and discriminant validity evidence. Journal of Personality and Social Psychology. 1980;39(3):472–80.
5. Cohen S, Kamarck T, Mermelstein R. A global measure of perceived stress. Journal of Health and Social Behavior. 1983;24(4):385–96.
6. Diener E, Emmons RA, Larsen RJ, Griffin S. The Satisfaction With Life Scale. Journal of Personality Assessment. 1985 Feb 1;49(1):71–5.
7. Greco V, Roger D. Coping with uncertainty: The construction and validation of a new measure. Personality and Individual Differences. 2001;31(4):519–34.
8. Adler NE, Epel ES, Castellazzo G, Ickovics JR. Relationship of subjective and objective social status with psychological and physiological functioning: preliminary data in healthy white women. Health Psychol. 2000 Nov;19(6):586–92.
9. Cohen J, Cohen P, West S, Aiken L. Applied Multiple Regression/Correlation Analysis for the Behavioral Sciences [Internet]. 3rd ed. Routledge; 2013 [cited 2021 Mar 25]. Available from: <https://www.taylorfrancis.com/books/9780203774441>
10. White H. A heteroskedasticity-consistent covariance matrix estimator and a direct test for heteroskedasticity. Econometrica: Journal of the Econometric Society 1980 May; 48(4): 817-838.
11. Baumsteiger R, Siegel JT. Measuring prosociality: The development of a Prosocial Behavioral Intentions Scale. Journal of Personality Assessment. 2019;101(3):305–14.
